# Artemether ameliorates type 1 diabetic liver injury alongside the associated defects in mitochondrial ultrastructure and central carbon metabolism

**DOI:** 10.1371/journal.pone.0348214

**Published:** 2026-04-29

**Authors:** Qike Fu, Jiaxin Li, Xiufen Gu, Yating Zhang, Pengxun Han, Xuewen Yu, Mumin Shao, Huili Sun, Yuchun Cai

**Affiliations:** 1 Department of Nephrology, Shenzhen Traditional Chinese Medicine Hospital Affiliated to Nanjing University of Chinese Medicine, Shenzhen, Guangdong, China; 2 Department of Nephrology, Shenzhen Traditional Chinese Medicine Hospital, The Fourth Clinical Medical College of Guangzhou University of Chinese Medicine, Shenzhen, Guangdong, China; 3 Department of Pathology, Shenzhen Traditional Chinese Medicine Hospital, The Fourth Clinical Medical College of Guangzhou University of Chinese Medicine, Shenzhen, Guangdong, China; Rutgers: Rutgers The State University of New Jersey, UNITED STATES OF AMERICA

## Abstract

Type 1 diabetes mellitus (T1DM) is characterized by autoimmune-mediated destruction of pancreatic islet β-cells and the resultant absolute insulin deficiency, which leads to systemic metabolic dysregulation. Hepatic injury has emerged as a clinically significant complication of T1DM; however, no targeted therapeutic intervention is currently available. Artemether (Art), a methyl ether derivative of artemisinin, has shown potential in ameliorating hyperglycemia, but its efficacy in mitigating T1DM-associated hepatic dysfunction remains insufficiently elucidated. This study comprehensively evaluates the hepatoprotective effects of Art in a murine model of T1DM, with particular emphasis on mitochondrial structural integrity and the regulation of glucose and lipid metabolism. Hepatic function was assessed through histopathological evaluation, ultrastructural examination of mitochondria via transmission electron microscopy, and molecular analysis of gene and protein expression levels. Metabolic intermediates associated with glucose and lipid metabolic pathways were quantitatively analyzed using ultra-high-performance liquid chromatography coupled with triple quadrupole tandem mass spectrometry (UPLC-QQQ-MS/MS). Administration of Art significantly attenuated both diabetic manifestations and liver injury. Importantly, Art preserved mitochondrial morphology, restored the expression of some proteins related to the respiratory chain complex, and downregulated indicators of hepatic fatty acid β-oxidation, upregulated markers of de novo fatty acid synthesis, normalized intrahepatic triglyceride concentrations, and reduced expression of key molecules involved in gluconeogenesis and glycogenolysis. These findings indicate that Art confers protective effects against liver injury in T1DM through coordinated modulation of mitochondrial function and key metabolic pathways at transcriptional, translational, and metabolic intermediate levels.

## 1. Introduction

Diabetes mellitus is a chronic and complex disease. It is mainly characterized by glucose metabolism disorders, which are caused by either absolute or relative insulin deficiency or reduced sensitivity of target cells to insulin. Type 1 diabetes mellitus (T1DM), specifically, is a chronic condition caused by the autoimmune destruction of pancreatic β cells. It is predicted that by 2045, the number of adults with diabetes worldwide will reach 783 million [1]. Current research on diabetic complications mainly focuses on nephropathy, retinopathy, neuropathy, cardiovascular diseases, and vasculopathy [2]. Diabetes is closely related to liver diseases such as non-alcoholic fatty liver, liver cirrhosis, and liver cancer. Numerous studies have shown that there are abnormal liver functions in T1DM mice [3]. Hepatic metabolic disorders are one of the pathological characteristics in patients with T1DM. Insulin resistance and disorders of glucose metabolism can lead to liver dysfunction [4]. Liver injury in type T1DM is often overlooked due to its atypical clinical symptoms. Nevertheless, with the increase in the mortality rate of end-stage liver disease among diabetic patients, it has gradually received more attention nowadays.

Artemisinin and its derivatives possess functions including anti-inflammation, immunomodulation, and anti-fibrosis [5,6]. Artemether (Art), as a derivative of artemisinin, also exhibits a series of beneficial biological activities. It can alleviate diabetes by reducing insulin resistance, improving the immune microenvironment, and restoring the function of pancreatic islet cells [7]. Studies have shown that in db/db mice and streptozotocin-induced T1DM mice, Art can reduce blood glucose levels and increase serum insulin levels, demonstrating the potential for therapeutic utility in treating islet dysfunction and improving insulin sensitivity [8,9]. Moreover, it also has a beneficial effect in improving diabetes complications such as diabetic nephropathy and non-alcoholic fatty liver disease [10,11].

In previous studies, it has been found that Art has the effect of improving hepatic glucose metabolism in T1DM and can enhance the function of hepatic mitochondria [12]. Given the close association between glucose metabolism and lipid metabolism in the body and considering that mice with T1DM often suffer from lipid metabolic disorders, this study further focuses on the effect of Art on hepatic lipid metabolism in T1DM mice. In this study, using T1DM mice as the research subjects, we aimed to explore the specific pathways through which Art ameliorates liver injury and modulates glucose and lipid metabolism in T1DM.

## 2. Materials and methods

### 2.1. Animals

Male C57BL/6J mice at the specific pathogen-free (SPF) grade were selected for the experiment. All these mice were purchased from the Guangdong Medical Experimental Animal Center and were housed in the SPF-grade animal facility of the Animal Experiment Center at the Shenzhen Graduate School of Peking University. All diets (routine diet for Control and T1D groups, medicated diet for T1D+Art group) were provided by Beijing Keao Xieli Feed Co.,Ltd. (China, Cat No,2112/2151) and were formulated to ensure consistent nutrient sources and calorie content. The mice had free access to food and water. The drinking water and bedding materials were replaced regularly to ensure an ample supply of drinking water and maintain the bedding in a dry and clean condition. The temperature of the rearing environment was controlled within the range of 20–23 °C, the relative humidity was kept at 50–60%, the light-dark cycle was set as 12 hours of light and 12 hours of darkness, and there was good ventilation. The animal studies were approved (approval no. 20190301011) by the guidelines of the Institutional Animal Care and Use Committee at Guangzhou University of Chinese Medicine (Guangzhou, China) and conducted in accordance with the Laboratory Animal Care Principles of the National Institutes of Health and the 3R principles (Replacement, Reduction, and Refinement).

### 2.2. Group modeling and drug intervention

**Fig 1:**
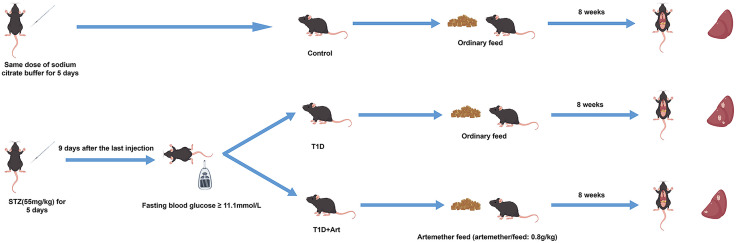
Sketch map which showed the process of the animal experiment.

Male C57BL/6J mice were randomly divided into three groups: the Control group, the type 1 diabetes mellitus group (T1D group), and the artemether group (T1D+Art group). Mice assigned to T1D group and T1D+Art group were subjected to a five consecutive-day intraperitoneal injection of streptozotocin (STZ, 55 mg/kg body weight, dissolved in citrate buffer) to establish a type 1 diabetes mellitus (T1DM) mouse model. Nine days after the last STZ injection, blood samples were collected from the tail veins of all mice to measure the fasting blood glucose level. The fasting blood glucose level of ≥ 11.1 mmol/L was regarded as the criterion for successful model establishment. After the setting up of the mouse model, the Control group and the T1D group mice were fed with a routine diet. The T1D+Art group was fed with a medicated diet (artemether/diet: 0.8 g/kg) (Chengdu ConBon Bio-tech Co., Ltd, China), and the total duration of drug intervention was 8 weeks(Fig 1). All operations and experimental procedures complied with the Regulations on the Management of Laboratory Animals in Guangdong Province.

### 2.3. Tissue preparation

Upon completion of the intervention, the mice were anesthetized with isoflurane for blood sample collection, followed by euthanasia via an overdose of isoflurane. Subsequently, the liver tissues were dissected and weighed. A part of the liver tissue was fixed in 10% formalin solution for histopathological assessment and immunohistochemical staining. The rest of the tissues were immediately snap-frozen in liquid nitrogen and stored at −80°C for subsequent analyses.

### 2.4. Biochemical assay

After the mice were fasted for 6 hours while having free access to water, a portable blood glucose meter (Roche, Basel, Switzerland) was employed to detect their fasting blood glucose level by collecting blood from the tail vein of the mice. After blood collection, the whole blood was drawn using a glycated hemoglobin analyzer (Primus, Kansas City, MO, USA) for the detection of glycosylated hemoglobin A1c (HbA1c). The concentration of serum alanine transaminase (ALT), aspartate transaminase (AST), and triglyceride (TG) were detected using an automated biochemical analyzer (Roche, Basel, Switzerland). Liver triglyceride content was measured according to the manufacturer’s instructions of the TG content determination kit (Solarbio, Beijing, China).

### 2.5. ELISA

Serum insulin was detected according to the instructions of the ELISA kit (Merck Millipore, Danvers, MA, USA). The serum insulin concentrations were calculated according to the formula and the optical density values of each sample.

### 2.6. Histopathological analysis

Paraffin sections of 3 μm thickness liver tissues were stained with hematoxylin-eosin (H&E) (Solarbio, China) and then sealed with neutral resin. After completing the above steps, the sections were scanned using the Digital Slide Scanner (3DHistech Ltd, Hungary).

### 2.7. Transmission electron microscopy

Samples of 1 mm³ liver tissue were fixed in glutaraldehyde fixative. After washing, the samples were fixed again, dehydrated at room temperature, infiltrated and embedded, followed by ultrathin sectioning and staining. The structural changes of mitochondria in the liver tissue were observed using a transmission electron microscope (JEM-1400, JEOL, Tokyo, Japan).

### 2.8. Immunohistochemical staining

The paraffin sections of liver tissues were deparaffinized and underwent antigen retrieval. After blocking, the sections were incubated with the primary antibodies (Table 1) overnight at 4 °C. On the next day, they were incubated with the secondary antibody, followed by color development. The color development time for the same indicators was controlled to be consistent. Then, the nuclei were stained with hematoxylin staining solution.

**Table 1:** Primary antibodies used for Western Blotting (WB) and immunohistochemistry (IHC).

Antibody Name	Company	Catalog Number
GSTP1	Proteintech	15902-1-AP
SOD2 (acetyl K68)	abcam	ab137037
ALDH2	Proteintech	68237-1-Ig
COXⅣ	CST	4844S
ND2	Proteintech	19704-1-AP
MTCO2	Proteintech	55070-1-AP
IDH3A	Proteintech	15909-1-AP
IDH3B	Proteintech	68199-1-Ig
ATGL	Proteintech	55190-1-AP
HSL	CST	18381S
FATP2	Proteintech	14048-1-AP
ACSS3	Proteintech	16204-1-AP
ACSM2A	Proteintech	22862-1-AP
CPT1A	Proteintech	15184-1-AP
CPT2	Proteintech	26555-1-AP
CACT	Proteintech	19363-1-AP
CROT	Proteintech	13543-1-AP
ACADM	Proteintech	55210-1-AP
ACLY	Proteintech	15421-1-AP
ACC	CST	3676S
FASN	Proteintech	10624-2-AP
GCK	Proteintech	19666-1-AP
PKLR	Proteintech	22456-1-AP
LDHA	Proteintech	19987-1-AP
PDK1	Enzo	ADI-KAP-PK112-F
P-PDH(Ser293)	CST	31866S
PDH	CST	2784S
P-PYGL(Ser15)	Abcam	ab227043
PYGL	Abcam	ab198268
PCK1	CST	12940S
PCK2	CST	6924S

### 2.9. Metabolites measurements

Mouse liver metabolites were analyzed using ultra-high-performance liquid chromatography (Shimadzu Corporation, Kyoto, Japan), coupled to the QTrap 5500 mass spectrometer (Sciex, Framingham, USA). Liver tissues (~ 12 mg) were extracted with 80% methanol (methanol: ultrapure water = 4:1[v/v]). After centrifugation, 10 µl of supernatant underwent hydrophobic interaction chromatography using a Luna Omega 1.6 μm Polar C18 reversed-phase column (Phenomenex, California, USA). Mobile phase A (10 mM ammonium acetate in ultrapure water, v/v) and mobile phase B (10 mM 90% acetonitrile/ammonium acetate [V/V]) were delivered at a flow rate of 0.3 mL/min. Gradient elution was as follows: from 2% to 60% B at 0–3.2 min; maintained at 60% from 3.21–3.5 min; and back to 2% B from 3.51–5 min to equilibrate the column before a new injection. Mass spectrometry data were acquired on a QTrap 5500 mass spectrometer using electrospray ionization in the negative ion multiple reaction monitoring mode with the following specifications: electrospray voltage of 5500 V, temperature of 500 °C, curtain gas at 40 psi, CAD gas at 12 psi, and gases 1 and 2 set at 50 psi each. The targeted metabolic profiling data was acquired by using MultiQuant 3.0.3 software (Sciex, Framingham, USA) to perform concentration calculations.

### 2.10. Western blot analysis

The proteins from liver tissues were extracted and their concentrations were determined. Separating gels of different concentrations were prepared according to the molecular weights of the target proteins, and stacking gels were also prepared. The samples and the marker were sequentially loaded into the electrophoresis lane and then subjected to electrophoresis. The proteins were transferred onto a PVDF membrane, which was then blocked. The membrane was cut according to the target molecular weights and placed into the corresponding incubation boxes containing the primary antibodies (Table 1), followed by overnight incubation on a shaker at 4 °C. Then, the membrane was incubated with the secondary antibody. The bands were soaked in the chemiluminescent solution for 30 seconds to 1 minute and then transferred to ChemiDoc Imaging System (Bio-Rad, Hercules, CA, USA) for exposure and development to obtain the corresponding band images. The ratio of the signal of the target band to that of the internal reference (ALDH2) band represents the expression level of the target protein.

### 2.11. Real-time fluorescent quantitative PCR

Total RNA was extracted from liver tissues using an RNA purification kit (Thermo Fisher Scientific, USA). First-strand cDNA synthesis was performed with oligo (dT)12–18 primers, dNTP Mix, ribonuclease inhibitor, DTT, and M-MLV reverse transcriptase (Thermo Fisher Scientific, USA). The reaction was conducted at 37 °C for 50 minutes, followed by termination at 70 °C for 15 minutes. Quantitative PCR was executed using SYBR Green PCR master mix (Thermo Fisher Scientific, USA) on the Applied Biosystems Quant Studio 5 (Thermo Fisher Scientific, USA). The amplification conditions were set to 95 °C for 5 minutes, followed by 45 cycles of 95 °C for 15 seconds, 55 °C for 15 seconds, and 72 °C for 20 seconds. The sequences of gene-specific primers (Sangon Biotech Co., China) were listed in Table 2. The relative expression level of the target gene mRNA was calculated using the 2^−ΔΔCt^ method, with ALDH2 as the housekeeping gene.

**Table 2:** The primer sequence for genes.

Gene	sequence5’-3’(Forward Primer)	sequence5’-3’(Reversed Primer)
*Idh3b*	AGGCACAAGATGTGAGGGTG	CAGCAGCCTTGAACACTTCC
*Nd1*	GGAACACTCCAAAAACAGACCT	CCACCACTGGGTATTGAGTAGAA
*Atgl*	TCCGTGGCTGTCTACTAAAGA	TGGGATATGATGACGTTCTCTCC
*Fatp2*	TCCTCCAAGATGTGCGGTACT	TAGGTGAGCGTCTCGTCTCG
*Acsl1*	ACCAGCCCTATGAGTGGATTT	CAAGGCTTGAACCCCTTCTG
*Cpt1a*	CTCCGCCTGAGCCATGAAG	CACCAGTGATGATGCCATTCT
*Cact*	GACGAGCCGAAACCCATCAG	AGTCGGACCTTGACCGTGT
*Crot*	GAACGGACATTTCAGTACCAGG	CTTCATTTGCGAATGGTTTCACT
*Acads*	GACTGGCGACGGTTACACA	GGCAAAGTCACGGCATGTC
*Acadl*	TGCCCTATATTGCGAATTACGG	CTATGGCACCGATACACTTGC
*Fasn*	AGGTGGTGATAGCCGGTATGT	TGGGTAATCCATAGAGCCCAG
*Gck*	TGAGCCGGATGCAGAAGGA	GCAACATCTTTACACTGGCCT
*Pdk1*	GCACTCCTTATTGTTCGGTGG	CGTCGCAGTTTGGATTTATGCT
*Pcx*	CAGTGGCTGTCTACTCGGAG	CCGCATCTACACCATTTTCCT
*Pck1*	CTGCATAACGGTCTGGACTTC	CAGCAACTGCCCGTACTCC

### 2.12. Statistical analysis

Statistical analyses and visual representations of the data were conducted utilizing GraphPad Prism version 10.1.2 (La Jolla, CA, USA). The results were expressed as the mean along with the standard deviation (SD) to provide a clearer understanding of the data distribution. To assess the statistical significance among the various groups, we employed Student’s t-test for two-group comparisons, and One-way ANOVA for comparisons involving multiple groups. Following this, Tukey’s multiple comparisons test was implemented as a post hoc analysis to further elucidate differences between group means. A *p*value of less than 0.05 was established as the threshold for determining statistical significance.

## 3. Results

### 3.1. Artemether ameliorated liver injury in T1DM mice

**Fig 2:**
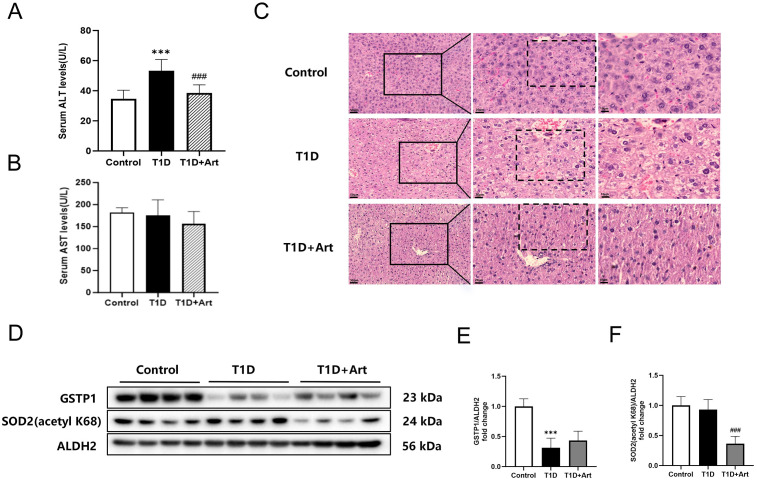
Artemether ameliorated liver injury in T1DM mice. **(A)** Serum ALT level. **(B)** Serum AST level. **(C)** HE staining of mouse liver sections, the scale bars are located in the lower left corner. The scale bars from left to right are 50 µm, 20 µm, and 10 µm. **(D-F)** Western blotting for GSTP1, and SOD2(acetyl K68) in the liver. ALDH2 serves as an internal reference. The results were indicated as the means ± SEMs (n = 6–8), ^***^p < 0.001 vs. Control group; ^###^*p* < 0.001 vs. the T1D group.

T1DM mice exhibited significant symptoms of diabetes, including polydipsia, polyphagia, polyuria, and weight loss. Treatment with Art alleviated polyuria in T1DM mice. Although there was a trend toward decreased water intake and stabilized food intake and body weight, these changes did not reach statistical significance compared with the T1D group. Additionally, Art treatment reduced fasting blood glucose and HbA1c levels in T1DM mice and increased serum insulin levels (Table 3). Compared with the control group, the serum ALT level in the T1D group mice was significantly increased. In contrast, when compared with the T1D group, the serum ALT level in the T1D+Art mice decreased significantly (Fig 2A). There were no significant changes in the serum AST levels among the mice in each group before and after the administration of the drugs (Fig 2B). HE staining of the liver with significant hepatocyte swelling and deformation were observed in T1DM mice, which improved after treatment with Art (Fig 2C). Western blot analysis showed that GSTP1 was downregulated in the liver of T1DM mice compared with control mice. After Art treatment, there was an upward trend. In contrast, the expression of SOD2 (acetyl K68) showed an increasing trend in the T1D group and was significantly reversed by Art treatment (Fig 2D-F).

**Table 3:** Art ameliorated diabetic symptoms and fasting blood glucose, HbA1c, and serum insulin levels in T1DM mice.

	Control	T1D	T1D+Art	Control vs. T1D	T1D vs. T1D+Art
				*p* value	*p* value
Water intake (ml/24h)	3.07 ± 0.61	23.64 ± 4.89***	20.91 ± 2.36	<0.001	0.203
Food intake (g/24h)	2.40 ± 0.23	4.64 ± 0.41***	4.98 ± 0.45	<0.001	0.699
Urine weight (g/24h)	1.27 ± 0.39	23.05 ± 3.60***	19.58 ± 1.86^#^	<0.001	0.025
Body weight (g)	27.13 ± 0.45	22.76 ± 0.52***	22.38 ± 0.76	<0.001	0.343
Fasting blood glucose (mmol/L)	5.86 ± 0.54	23.04 ± 3.21***	16.36 ± 1.85^###^	<0.001	<0.001
HbA1c (%)	4.31 ± 0.16	10.75 ± 0.54***	8.75 ± 0.45^###^	<0.001	<0.001
Serum insulin concentration (ng/ml)	3.44 ± 1.44	0.64 ± 0.29***	1.11 ± 0.32^#^	<0.001	0.018

The results were indicated as the means ± SEMs. (n = 6–8), ^***^p < 0.001 vs. control group; ^#^*p* < 0.05 and ^###^*p* < 0.001 vs. the T1D group.

### 3.2. Artemether restored the shape and structure of mitochondria in the liver of T1DM mice

**Fig 3:**
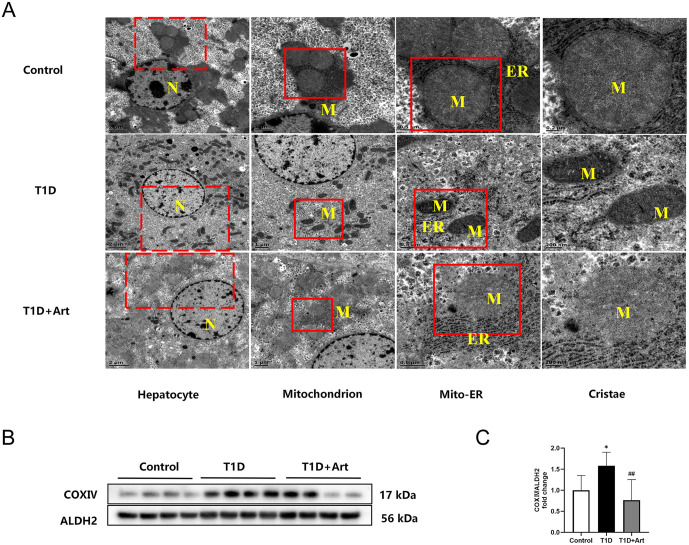
The effects of artemether on the structure of liver mitochondria. **(A)** The structure of mitochondria in the liver of mice under transmission electron microscopy, showing nuclei **(N)**, endoplasmic reticulum (ER) and mitochondria(M), the red dashed boxes represent the incomplete magnified view, while the red solid boxes denote the complete magnified view. The scale bars are located in the lower left corner. **(B-C)** Western blotting for COXⅣ in the liver. ALDH2 serves as an internal reference. The results were indicated as the means ± SEMs (n = 6-8). ^*^p < 0.05 vs. Control group; ^##^*p* < 0.01 vs. the T1D group.

Systemic diabetic manifestations and liver injury in T1DM mice prompt investigation into mitochondrial function, as mitochondria are central to hepatic energy metabolism and their structural damage is a key driver of diabetic liver injury. Fig 3A showed the structural characteristics of mitochondria in the liver of mice. The mitochondria in the liver of control mice were oval in shape, featuring closely arranged cristae, with mitochondria in close contact with the endoplasmic reticulum. In contrast, the mitochondria in the liver of T1DM mice exhibited irregular shape and disrupted cristae structure. The mitochondrion exhibited a loose association with an ER. In comparison, the T1DM+Art group displayed spherical mitochondria with closely arranged cristae, re-establishing connections with the endoplasmic reticulum. Compared to the control group, the expression levels of COXⅣ in the T1D group were elevated; however, after Art treatment, its expression levels decreased (Fig 3B and C).

### 3.3. Artemether ameliorates dysregulated expression of key molecules in hepatic energy metabolism of T1DM mice

**Fig 4:**
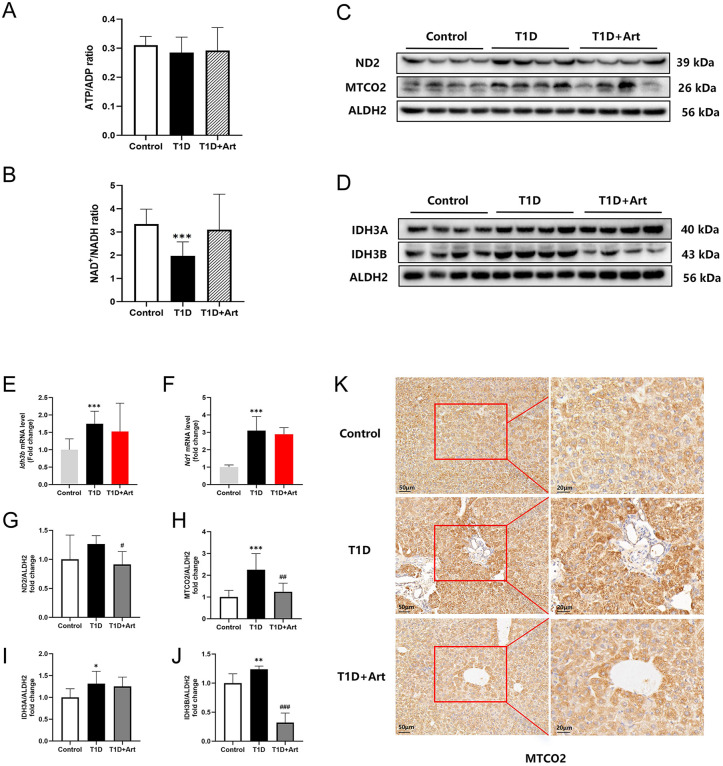
Artemether regulated mitochondrial function in the liver. **(A)** The ratio of ATP to ADP in the liver. **(B)** The ratio of NAD^+^ to NADH in the liver. (**E**) mRNA expression levels of *Idh3b* in the liver. (**F**) mRNA expression levels of *Nd1* in the liver. (**C**, **G**, **H)** Western blotting for ND2 and MTCO2 in the liver. **(K)** IHC for MTCO2 in liver, the scale bars are located in the lower left corner. (**D**, **I**, **J)** Western blotting for IDH3A and IDH3B in the liver. ALDH2 serves as an internal reference. The results are indicated as the means ± SEMs (n = 4-8), ^*^p < 0.05, ^**^p < 0.01, and ^***^p < 0.001 vs. Control group; ^#^*p* < 0.05, ^##^*p* < 0.01 and ^###^*p* < 0.001 vs. the T1D group.

Through the detection of energy metabolites, it was found that compared to the control group, the levels of ADP, and NADH elevated in the liver of T1DM mice, and these levels decreased after Art treatment (Table 4). However, further analysis revealed that Art tended to increase the reduced ratios of ATP/ADP and NAD ⁺ /NADH in the liver of T1DM mice, with no statistically significant difference observed. (Fig 4A and B). ND1 and ND2 are subunits of mitochondrial complex I, while MTCO2 is a subunit of mitochondrial complex IV. Compared to the control group, the mRNA and/or protein expressions of the ND1, ND2, and MTCO2 in the liver of T1DM mice increased; however, these expressions were reduced by the administration of Art (Fig 4C, F-H). IDH3A and IDH3B are two subunits of IDH3, which is the key enzyme in the TCA cycle. The results of WB showed that in the liver of T1DM mice, the protein expression of IDH3A and IDH3B increased, Art intervention reduced these two protein expressions to some degree (Fig 4D, E, I and J).

**Table 4:** The content of mitochondrial metabolites in the liver. The results were indicated as the means ± SEMs. (n = 6–8), *p < 0.05 and **p < 0.01 vs. Control group; #*p* < 0.05 vs. the T1D group.

Metabolites (ng/mg)	Control	1D	T1D+Art	Control vs. T1D	T1D vs. T1D+Art
				*p* value	*p* value
ATP	844.92 ± 189.46	1035.71 ± 321.05	855.55 ± 345.91	0.170	0.299
ADP	2613.84 ± 523.49	3584.65 ± 568.19**	2845.20 ± 645.99^#^	0.004	0.029
NAD+	2132.98 ± 22.81	2232.75 ± 583.72	1725.90 ± 535.64	0.705	0.092
NADH	602.47 ± 180.04	1252.25 ± 556.37**	718.84 ± 404.83^#^	0.007	0.046

### 3.4. Artemether prevented hepatic fat mobilization and fatty acid activation in T1DM mice

**Fig 5:**
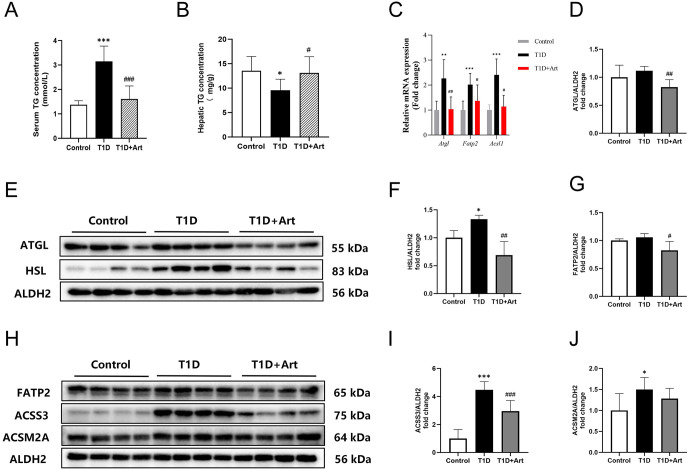
Artemether prevented hepatic fat mobilization and fatty acid activation, and regulated triglycerides in T1DM mice to normal levels. **(A)** Serum TG levels in the mice. **(B)** Hepatic TG levels in the mice. (**C**) mRNA expression levels of *Atgl*, *Fatp2* and *Acsl1* in the liver. **(D-F)** Western blotting for ATGL and HSL in the liver. **(G-J)** Western blotting for FATP2, ACSS3 and ACSM2A in the liver. ALDH2 serves as an internal reference. The results were indicated as the means ± SEMs(n = 4-8), ^*^p < 0.05, ^* *^p < 0.01 and ^***^p < 0.001 vs. Control group; ^*#*^*p* < 0.05, ^*##*^*p* < 0.01 and ^*###*^*p* < 0.001 vs. the T1D group.

Mitochondrial dysfunction in T1DM livers is closely associated with altered substrate utilization—diabetic conditions shift hepatic energy supply from glucose to fatty acids, overwhelming mitochondria with excessive fatty acid β-oxidation [13]. Thus, we further analyzed lipid and glucose metabolism to clarify how Art modulates metabolic substrates for mitochondrial protection. The results showed that the serum TG increased, and the liver TG decreased in the T1DM mice, while both liver and serum TG were reversed following Art treatment (Fig 5A and B). Compared to the control group, the mRNA expression levels of *Atgl*, *Fatp2* and *Acsl1* in the T1D group were elevated; and all decreased after Art treatment (Fig 5C). Further analysis of key proteins involved in fat mobilization (ATGL, HSL), transport and fatty acid activation (FATP2, ACSS3 and ACSM2A) through Western blotting revealed that, compared to the control group, the expression levels of HSL, ACSS3 andACSM2A were elevated in the liver of T1D mice. After treatment with Art, the expression levels of these proteins all showed a decrease to different degrees (Fig 5D-J).

### 3.5. Artemether inhibited liver fatty acid β oxidation in T1DM mice

**Fig 6:**
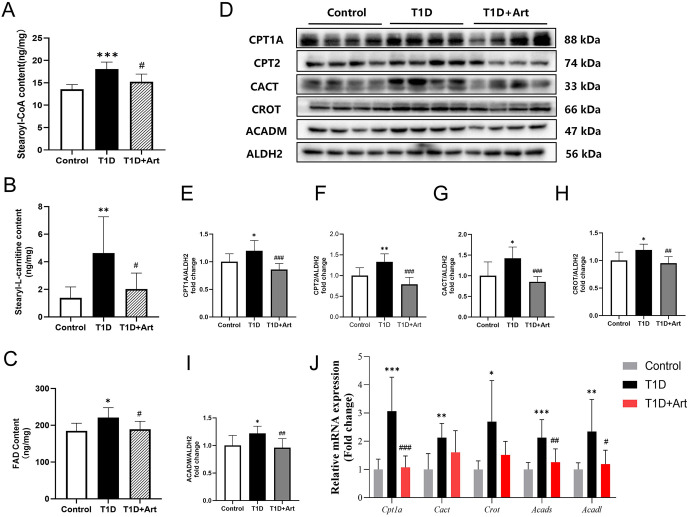
Artemether inhibited liver fatty acidβ oxidation in T1DM mice. **(A)** Hepatic stearoyl-CoA content in the mice. **(B)** Hepatic stearoyl-L-carnitine content in the mice. **(C)** Hepatic FAD levels in the mice. (**D**-**I**) Western blotting for CPT1A, CPT2, CACT, CROT, and ACADM in the liver. ALDH2 serves as an internal reference. (**J**) mRNA expression levels of *Cpt1a*, *Cact*, *Crot*, *Acads,* and *Acadl* in the liver. The results were indicated as the means ± SEMs (n = 4–8), ^*^p < 0.05, ^**^p < 0.01, and ^***^p < 0.001 vs. Control group; ^*#*^*p* < 0.05, ^*##*^*p* < 0.01, and ^*###*^*p* < 0.001 vs. the T1D group.

In the T1D group, the levels of stearoyl-CoA, stearyl-L-carnitine, and FAD were significantly elevated. After the intervention of Art, they were significantly lower compared with the T1D group (Fig 6A-C). RT-qPCR results indicated that Art could decrease the expression levels of mRNA related to fatty acid β-oxidation, including *Cpt1a*, *Cact*, *Crot*, *Acads*, and *Acadl* in the liver of T1D mice (Fig 6J). Western blot analysis revealed that compared to the control group, the expression levels of fatty acid β-oxidation related proteins CPT1A, CPT2, CACT, CROT, and ACADM were increased in the T1D group. Following Art treatment, the expression levels of these enzymes decreased (Fig 6D-I).

### 3.6. Artemether increased de novo fatty acid synthesis of hepatic fatty acids in the T1DM mice

**Fig 7:**
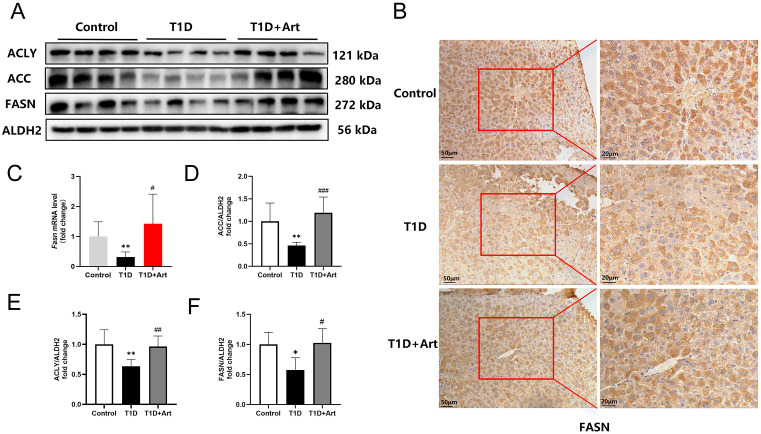
Artemether increased de novo synthesis of fatty acids in the liver of T1DM mice. (**A**) and **(D-F)** Western blotting for ACLY, ACC, and FASN in the liver. ALDH2 serves as an internal reference. **(B)** IHC for FASN in the liver, the scale bars are located in the lower left corner. (**C**) mRNA expression levels of *Fasn* in the liver. The results were indicated as the means ± SEMs (n = 4–8), ^*^p < 0.05 and ^**^p < 0.01 vs. Control group; ^*#*^*p* < 0.05, ^*##*^*p* < 0.01, and ^*###*^*p* < 0.001 vs. the T1D group.

Considering that de novo fatty acid synthesis is equally critical, we further assayed the corresponding key indicators (ACLY, ACCα, and FASN). Western blot analysis showed that ACLY, ACCα, and FASN protein levels were downregulated in the liver of T1DM mice compared with the control mice, while Art treatment significantly increased the expression levels of these proteins (Fig 7A, D-F). IHC results showed that the expression of FASN in the liver of T1DM mice was weakened and it rose after Art treatment (Fig 7B). RT-qPCR results demonstrated that Art could restore the mRNA expression levels of hepatic *Fasn* in the T1D group (Fig 7C).

### 3.7. Artemether promoted the oxidative utilization of glucose in the liver of T1DM mice

**Fig 8:**
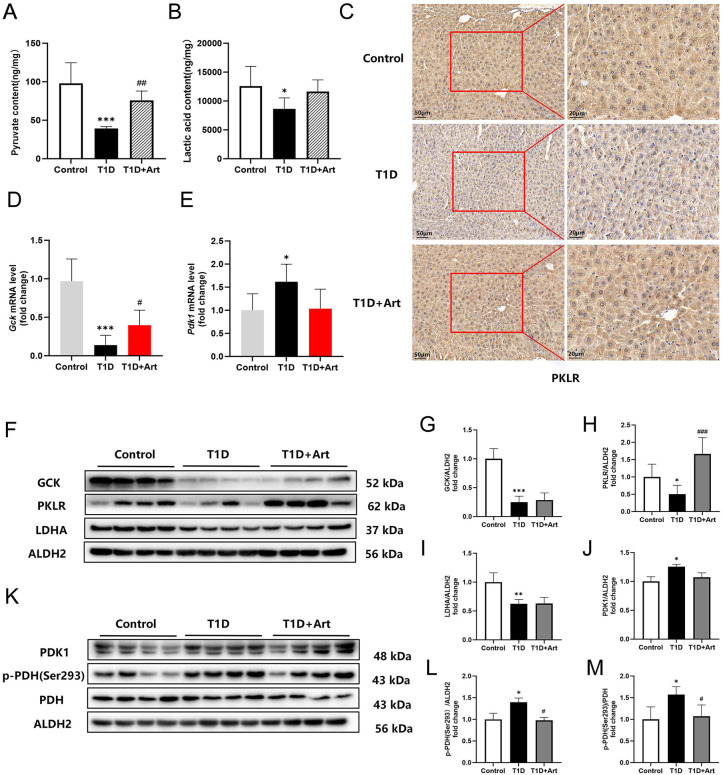
Artemether promoted the oxidative utilization of glucose in the liver of T1DM mice. **(A)** Hepatic pyruvate content in T1DM mice. **(B)** Hepatic lactic acid content in T1DM mice. **(C)** IHC for PKLR in the liver, the scale bars are located in the lower left corner. (**D**) mRNA expression levels of *Gck* in the liver. (**E**) mRNA expression levels of *Pdk1* in the liver. (**F**-**I**) Western blotting for GCK, PKLR and LDHA in the liver. **(J-M)** Western blotting for PDK1, p-PDH(Ser293), and PDH in the liver. ALDH2 serves as an internal reference. The results were indicated as the means ± SEMs (n = 4–8) ^*^p < 0.05, ^**^p < 0.01 and ^***^p < 0.001 vs. Control group; ^*#*^*p* < 0.05, ^*##*^*p* < 0.01, and ^*###*^*p* < 0.001 vs. the T1D group.

As products of glycolysis, the contents of hepatic pyruvate and lactic acid in the T1D group were significantly lower than those in the control group, and they increased after Art treatment (Fig 8A and B). IHC results showed that the expression of PKLR in the liver of T1DM mice was weakened, and it rose after Art treatment (Fig 8C). GCK and PKLR are the glycolysis rate-limiting enzymes in the liver. RT-qPCR results indicated that Art could increase the mRNA expression level of *Gck* and *Pdk1* in the liver of T1D mice (Fig 8D and E). Under anaerobic conditions, LDHA facilitates the conversion of pyruvate into lactate. Western blot analysis revealed that the protein expression levels of GCK, PKLR and LDHA in the liver of the T1D group decreased compared to the control group, and Art could increase the level of PKLR in the liver of T1DM mice (Fig 8F-I). Alternatively, pyruvate can translocate from the cytoplasm into the mitochondrial matrix, where it undergoes oxidative decarboxylation by pyruvate dehydrogenase (PDH) in an aerobic environment, resulting in the formation of acetyl coenzyme A. PDK1 can phosphorylate PDH, leading to its activity being inhibited. Western blot analysis showed a significant increase in hepatic PDK1 and p-PDH (Ser293) levels in the T1D group, while the levels of both two proteins decreased after Art intervention (Fig 8J-M).

### 3.8. Artemether decreased hepatic glycogenolysis and gluconeogenesis in T1DM mice

**Fig 9:**
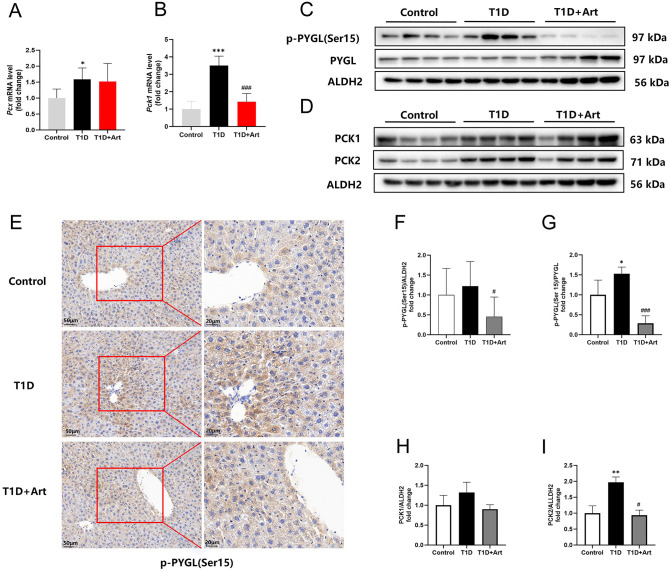
Artemether inhibited glycogenolysis and gluconeogenesis in T1DM mice. (**A**) mRNA expression levels of *Pcx* in the liver. (**B**) mRNA expression levels of *Pck1* in the liver. (**C**, **F**, **G)** Western blotting for p-PYGL(Ser15) and PYGL in the liver. (**D**, **H**, **I)** Western blotting for PCK1 and PCK2 in the liver. **(E)** IHC for p-PYGL(Ser15) in the liver, the scale bars are located in the lower left corner. ALDH2 serves as an internal reference. The results were indicated as the means ± SEMs (n = 4–8), ^*^p < 0.05, ^**^p < 0.01, and ^***^p < 0.001 vs. Control group; ^#^*p* < 0.05 and ^###^*p* < 0.01 vs. the T1D group.

PCX, PCK1, and PCK2 are key enzymes in gluconeogenesis. RT-qPCR analysis indicated an increase in *Pcx* and *Pck1* expression in the liver of T1D mice (Fig 9A and B). When PYGL is activated by phosphorylation, it promotes the degradation of glycogen, leading to an increase in blood glucose levels. IHC and WB results showed that compared to the control group, the T1D group exhibited an increase in p-PYGL protein levels, which significantly decreased after Art intervention (Fig 9C, E-G). Western blot analysis showed a significant increase in the hepatic PCK2 level in the T1D group; however, the level decreased after Art intervention, and the protein expression level of PCK1 was not statistically significant. (Fig 9D, H and I).

## 4. Discussion

**Fig 10:**
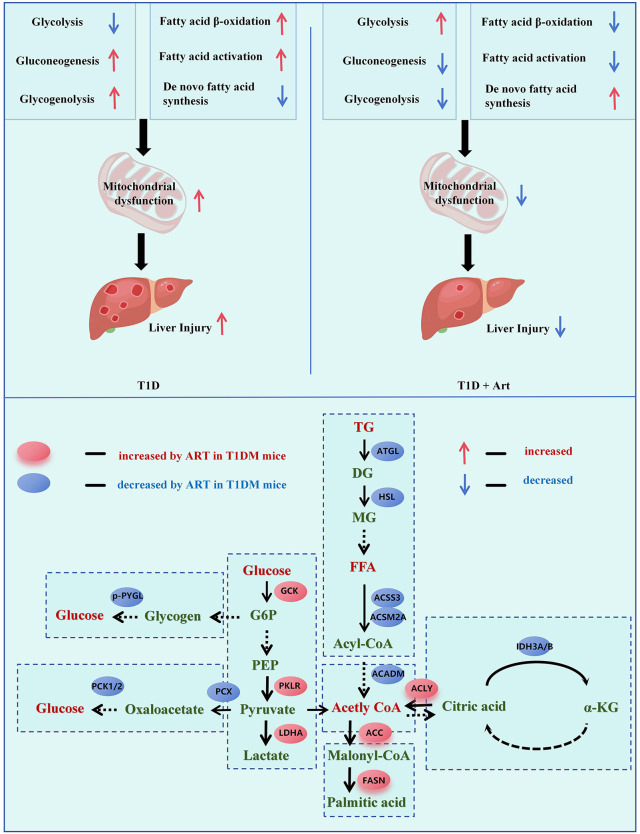
The schematic diagram illustrates hepatic glucose metabolism, lipid metabolism, and mitochondrial metabolism in the context of Art intervention in T1DM mice. In T1D, glycolysis is reduced, while gluconeogenesis, glycogenolysis, fatty acid β-oxidation/activation are enhanced, and de novo fatty acid synthesis is inhibited—leading to mitochondrial dysfunction and liver injury. In contrast, T1D+Art intervention increases glycolysis, decreases gluconeogenesis/glycogenolysis, inhibits fatty acid β-oxidation/activation, and promotes de novo fatty acid synthesis, thereby alleviating mitochondrial dysfunction and reducing liver injury. Art modulates key enzymes/metabolites in glucose and lipid metabolism. For glucose metabolism, Art affects glycogenolysis (via p-PYGL), gluconeogenesis (via PCK1/2, PCX), and glycolysis (via GCK, PKLR, LDHA). For lipid metabolism, it influences triglyceride (TG) hydrolysis (via ATGL, HSL), fatty acid activation (via ACSS3, ACSM2A), de novo fatty acid synthesis (via ACLY, ACC, FASN), and TCA cycle (via IDH3A/B). Red/blue indicators respectively show Art-upregulated/downregulated molecules in T1D mice, highlighting Art’s role in restoring metabolic homeostasis and mitigating T1D-related liver injury.

In this study, we investigated the protective effects of Art on the liver in T1DM and explored its potential mechanisms related to mitochondrial function and glucose-lipid metabolism. The results indicated that Art provides a certain degree of protective effect on liver function in STZ-induced T1DM mice. Furthermore, Art regulates hepatic energy metabolism. The observed effects include improving the ultrastructure of mitochondria and the expression of key subunits of respiratory chain complexes, inhibiting fatty acid β-oxidation, and promoting the oxidation and utilization of glucose. (Fig 10).

As the energy supply factories of cells, mitochondria’s inner membrane electron transport chain is the primary site for ATP production. Dysregulation of NAD+ homeostasis can result in impaired energy metabolism and heightened oxidative stress [14]. The ATP/ADP and NAD^+^/NADH ratios are important indicators of mitochondrial function [15]. Although ATP and NAD^+^ content increased in the diabetic hepatocytes, the ATP/ADP and NAD + /NADH ratios decreased to some extent, while Art could raise them in our experiment. These results indicated that Art could partly restore mitochondrial function, which might be the underlying mechanism for the amelioration of the diabetic hepatocytes injury.

Mitochondrial morphology is important for its function. In general, fragmented mitochondria are bioenergetically impaired, while hyperfused mitochondria appear to have high bioenergetic capacity [16]. On the other hand, mitochondrial cristae structure and density direct the oxidative phosphorylation output [17]. In our study, the increased small fragmented mitochondria and irregular cristae in the liver cells of type 1 diabetic mice suggested that ATP production capacity was relatively insufficient relatively for the mitochondria in the diabetic hepatocytes. Belosludtsev et al. investigators had found that the liver mitochondria of T2DM mice exhibited stress-induced fission and destruction of the cristae structures [18]. Additionally, MAM regulates mitochondrial fusion and fission, as well as mitochondrial respiratory supercomplexes assembly factor 1 to maintain mitochondrial homeostasis for ATP production [19,20]. In our experiment, disturbed MAM was obvious in the diabetic hepatocytes, which could lead to dysfunction of mitochondria. Art treatment normalized mitochondrial structure and MAM, which could be associated with higher ATP production efficiency. High glucose can lead to destruction of liver MAM integrity by impacting protein phosphatase 2A activity [20,21].

The TCA cycle and OXPHOS are the two key steps in ATP production in mitochondria. The TCA cycle is the hub of the three major metabolic pathways in the cell, which provide a bulk of reducing equivalent for electron transfer and OXPHOS [22,23]. To investigate the potential mechanisms of Art on mitochondrial ATP production, we examined some mitochondrial respiratory chain complex-related proteins and rate-limiting enzymes in the TCA cycle. In our study, the expression levels of mitochondrial respiratory chain subunits (ND2, MTCO2, ND1), and TCA cycle key enzyme IDH3 in the livers of T1DM mice were found to be increased. Art intervention reversed this dysregulated expression. The upregulation of these mitochondrial proteins in T1DM mice likely represents a cellular compensatory adaptation to metabolic disturbance. Diabetic conditions induce oxidative stress and cristae damage, which may reduce the catalytic efficiency of individual complexes/enzymes. Cells upregulate protein expression to maintain total activity, but this compensation is insufficient—as evidenced by ATP/ADP ratio and disrupted cristae structure.

Art’s downregulation of these proteins is not “inhibition” but “restoration of normal expression levels”. After Art treatment, mitochondrial cristae structure is repaired, and catalytic efficiency of complexes/enzymes tends to be recovered. Thus, the need for compensatory overexpression is eliminated, and protein levels return to control levels. These changes are consistent with the manifestation that mitochondrial and TCA cycle function may be regulated.

The TCA cycle and electron transfer enhanced in the diabetic hepatocyte mitochondria maybe associated with the energy substrates supply mode switching. In this study, the liver of T1DM mice exhibited diminished glycolysis, while lipolysis was initiated and fatty acid β-oxidation was enhanced. Concurrently, de novo fatty acid synthesis was reduced in diabetic hepatocytes. These findings suggest that under T1DM conditions, the energy supply mode in the liver has undergone changes. Oxidative phosphorylation using non-esterified fatty acids as substrates has replaced the energy supply mode that relied on glucose. Excessive free acid β-oxidation overwhelm mitochondrial capacity [13,24]. In our experiment, free fatty acid transport protein (FATP2), acyl-COA synthetase for activation of fatty acids (ACSS3 and ACSS2A), mitochondrial free fatty acid transport protein (CPT1A, CPT2, CACT, CROT) and acyl-CoA dehydrogenase (ACADM, ACADS, ACADL), as well as free fatty acid β-oxidation associated related metabolite (stearoyl-CoA, stearoyl-L-Carnitine and FAD) were all increased in the diabetic hepatocytes. Additionally, hepatic enhanced lipolysis (ATGL, HSL) and attenuated lipogenesis (ACLY, ACCα, FASN) accelerated the accumulation of free fatty acid content in the diabetic mice, and furtherly drove free fatty acid β-oxidation while overwhelming mitochondrial burden. Dewidar B et al. found that hepatic free fatty acid oxidation associated respiration doubled in type 1 diabetic mice [25]. The lipid synthesis in T1DM patients and mouse model were reduced [26–28]. Art can effectively inhibit the excessive activation and transport of fatty acids in the liver of T1DM mice; prevent excessive fatty acids from entering the mitochondria; attenuate hepatic lipolysis, enhance the liver’s ability to synthesize fatty acids; thereby alleviate the metabolic stress on the hepatocyte mitochondria.

The abnormal free fatty acids metabolism in the liver of T1DM is probably relevant to the glucose utilization disability. The liver serves as an important organ for controlling glucose homeostasis, regulating glycolysis, gluconeogenesis, glycogenesis, and glycogenolysis. Previous research demonstrated that hepatic glycolysis is weakened [29–31], and gluconeogenesis is enhanced in both T1DM and T2DM [32–34]. In our study, the difference in key enzymes of glycolysis (GCK and PKLR), key enzymes of gluconeogenesis (PCK1 and PCK2), LDHA, p-PDH(Ser293), as well as pyruvate and lactic acid content in the liver of three group of mice indicated that glycolysis, glucose aerobic oxidation and anaerobic oxidation were weakened while gluconeogenesis was enhanced in the liver of T1DM mice; this can be reversed by Art treatment.

Art exhibited hepatoprotective effects in T1DM mice, which might be achieved through mechanisms related to the regulation of liver mitochondrial function. This effect may be associated with the correction of energy substrate preference from fatty acids to glucose observed after Art treatment (Fig 10). Although we quantified many key enzymes and intermediate metabolites involved in glucose and free fatty acid metabolism in the liver of T1DM mice, this study still has some limitations. A limitation of the present study lies in the lack of direct functional measures to fully confirm the mechanistic claims related to mitochondrial activity and metabolic pathway regulation. Moreover, a single metabolite may participate in multiple metabolic pathways, making it challenging to determine its source and fate. Further studies are needed to clarify the mechanism of Art’s regulation of hepatic metabolism in T1DM through targeted intervention experiments and metabolic flux analyses of glycolysis, β-oxidation, and de novo lipogenesis.

## Supporting information

S1 File Raw gel blot images. (PDF)

S2 File Data for figures. (XLSX)

S3 File Data for tables. (XLSX)

S4 File HE, TEM and IHC. (PDF)
