# Supplementary material for: Artemether ameliorates type 1 diabetic liver injury alongside the associated defects in mitochondrial ultrastructure and central carbon metabolism
Source: PLoS One. 2026 Apr 29;21(4):e0348214. doi: 10.1371/journal.pone.0348214 (PMC13127903; doi:10.1371/journal.pone.0348214)
Supplement: S1 File — (PDF) [file pone.0348214.s001.pdf]

Supplementary Raw Western Blot Data

Panel 1 represents western blot quantitative analysis shown in Fig 2D

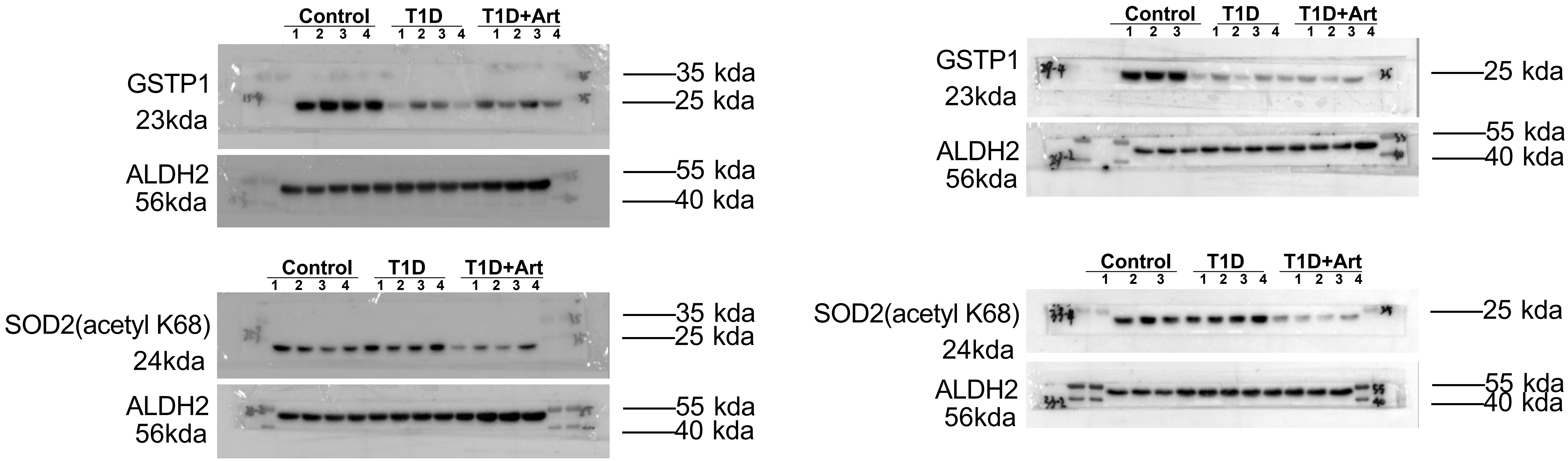

Marker: PageRuler$_{mw}$ Prestained Protein Ladder, 10 to 180 kDa, 26616, Thermo Scientific.

Supplementary Raw Western Blot Data

Panel 1 represents western blot quantitative analysis shown in Fig 3B

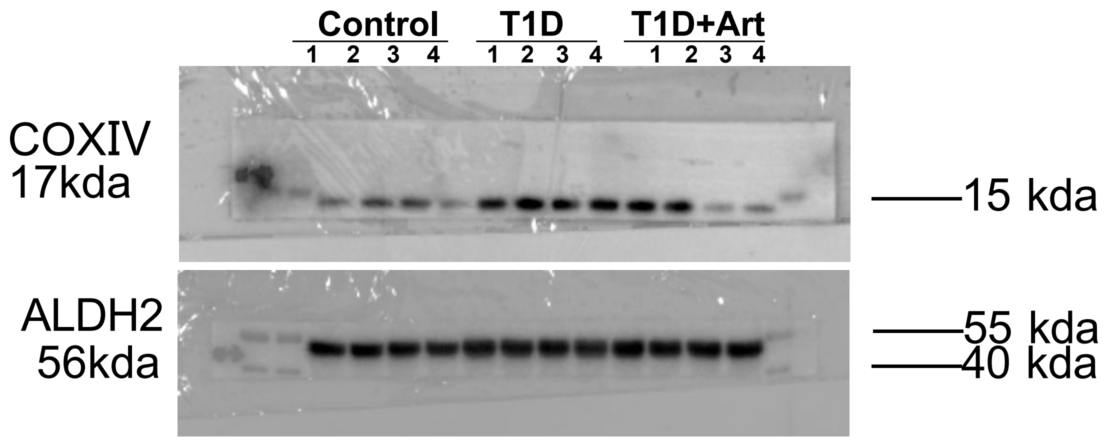

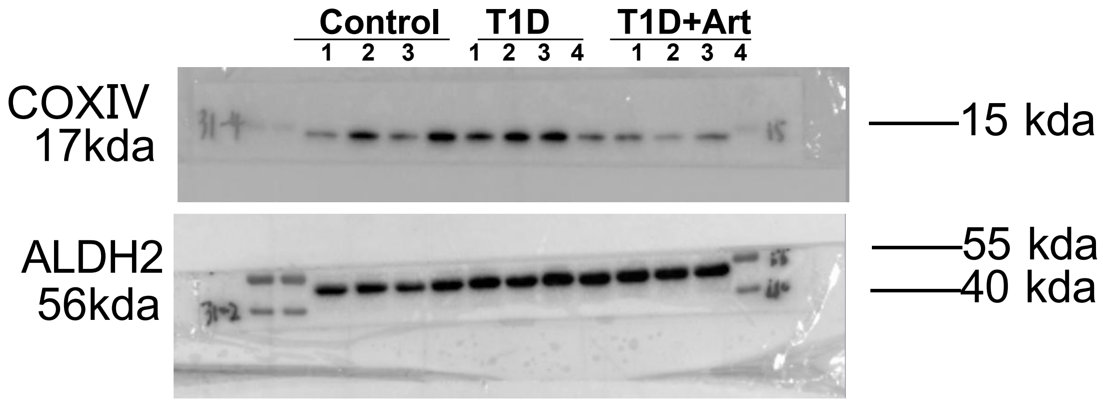

Marker: PageRuler$_{mw}$ Prestained Protein Ladder, 10 to 180 kDa, 26616, Thermo Scientific.

Supplementary Raw Western Blot Data

Panel 1 represents western blot quantitative analysis shown in Fig 4C

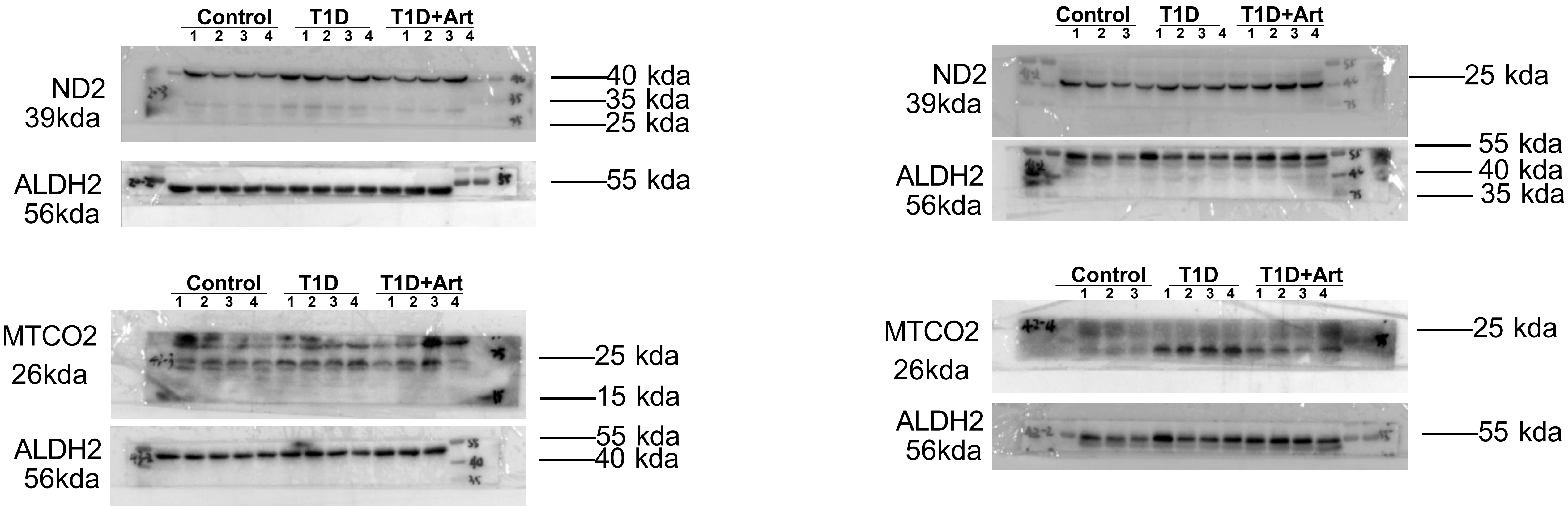

Marker: PageRuler$_{mw}$ Prestained Protein Ladder, 10 to 180 kDa, 26616, Thermo Scientific.

Supplementary Raw Western Blot Data

Panel 1 represents western blot quantitative analysis shown in Fig 4D

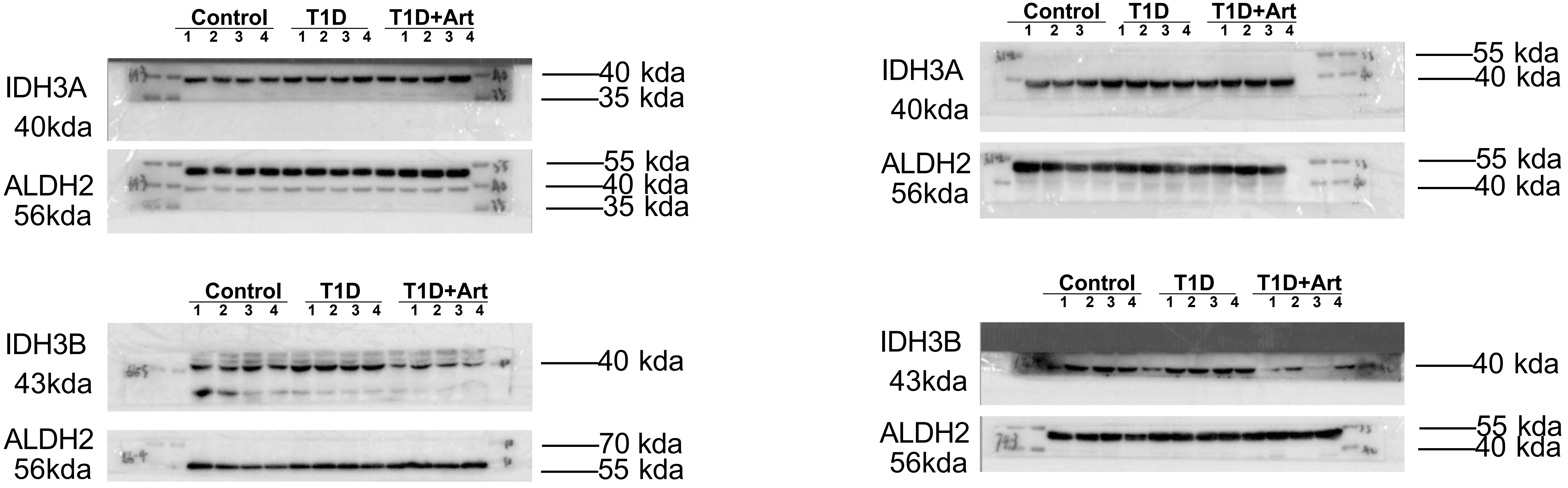

Marker: PageRuler$_{mw}$ Prestained Protein Ladder, 10 to 180 kDa, 26616, Thermo Scientific.

Supplementary Raw Western Blot Data

Panel 1 represents western blot quantitative analysis shown in Fig 5E

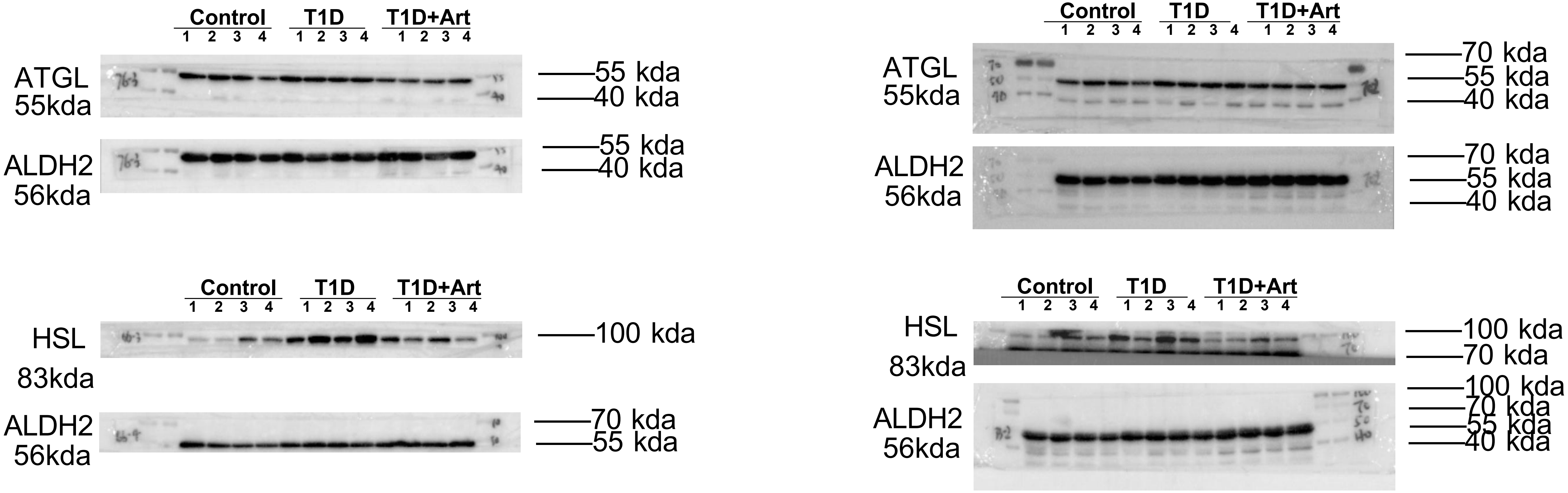

Marker: PageRuler$_{mw}$ Prestained Protein Ladder, 10 to 180 kDa, 26616, Thermo Scientific.

Supplementary Raw Western Blot Data

Panel 1 represents western blot quantitative analysis shown in Fig 5H

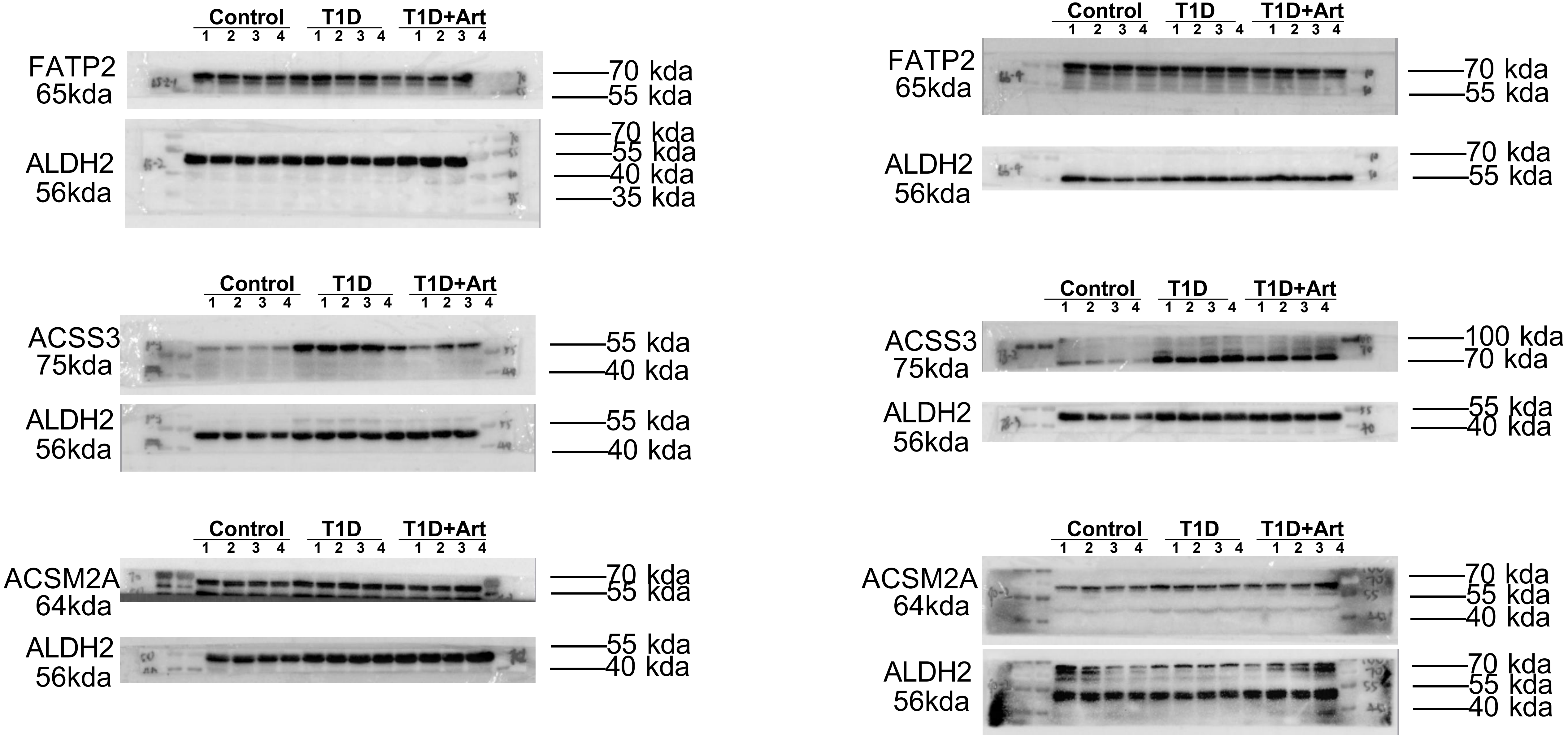

Marker: PageRuler mw Prestained Protein Ladder, 10 to 180 kDa, 26616, Thermo Scientific.

Supplementary Raw Western Blot Data

Panel 1 represents western blot quantitative analysis shown in Fig 6D

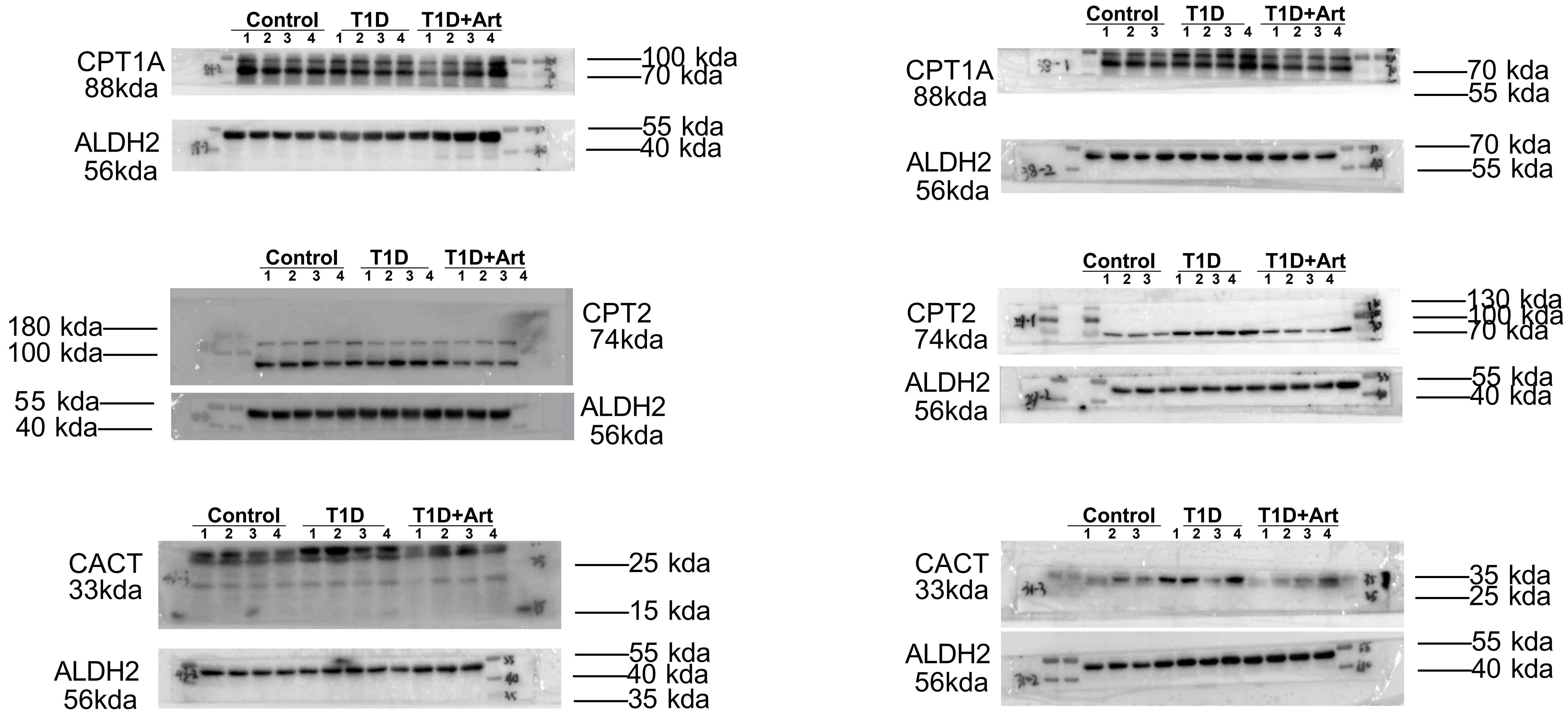

Marker: PageRuler$_{mw}$ Prestained Protein Ladder, 10 to 180 kDa, 26616, Thermo Scientific.

Supplementary Raw Western Blot Data

Panel 1 represents western blot quantitative analysis shown in Fig 6D

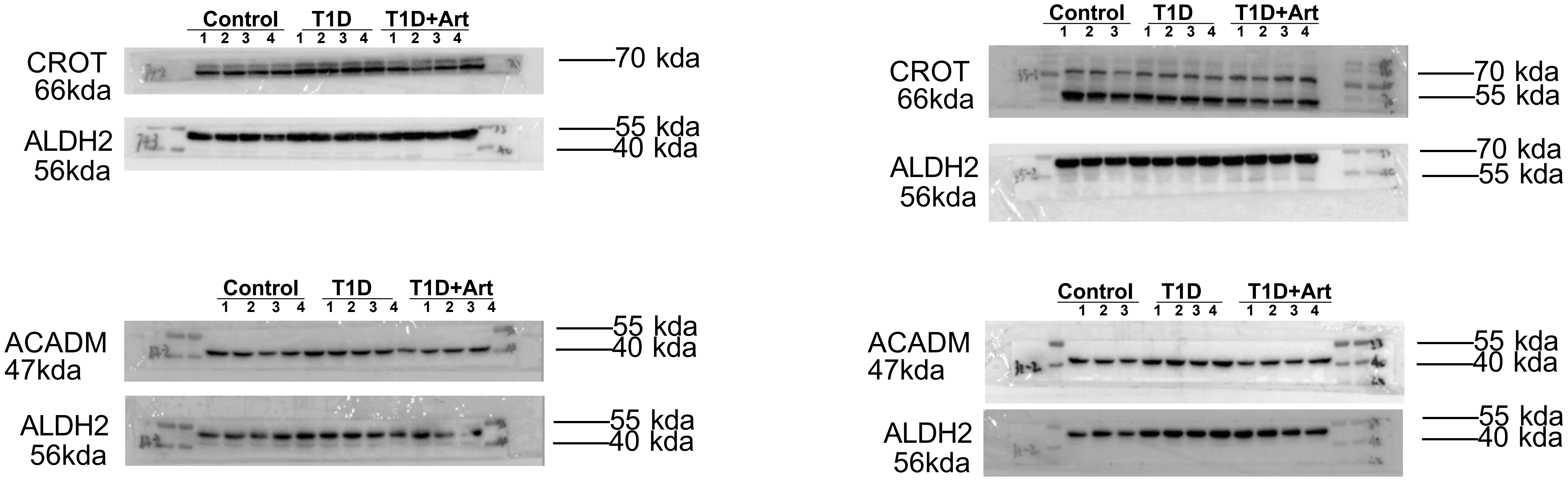

Marker: PageRuler$_{mw}$ Prestained Protein Ladder, 10 to 180 kDa, 26616, Thermo Scientific.

Supplementary Raw Western Blot Data

Panel 1 represents western blot quantitative analysis shown in Fig 7A

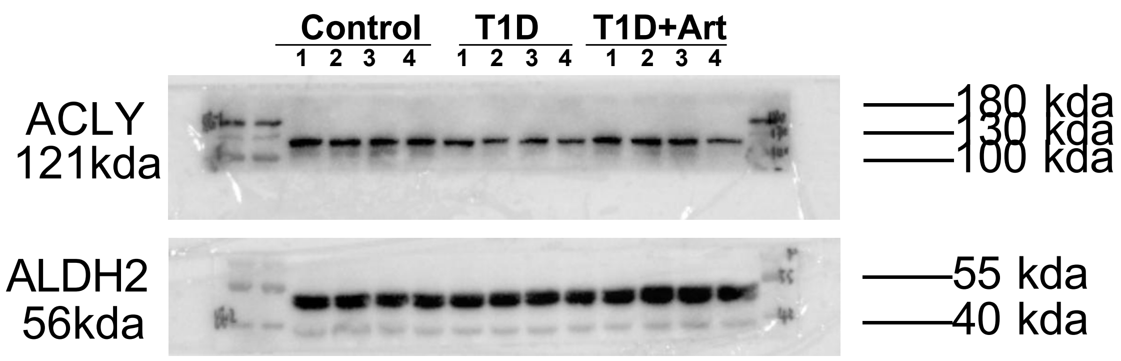

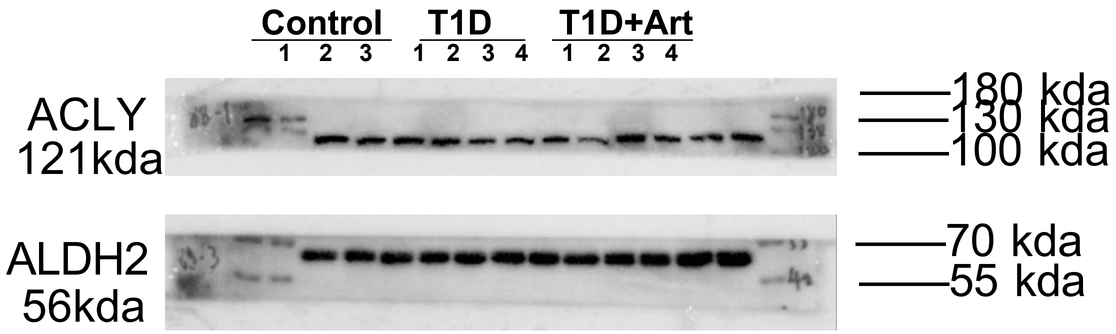

Marker: PageRuler$_{mw}$ Prestained Protein Ladder, 10 to 180 kDa, 26616, Thermo Scientific.

Supplementary Raw Western Blot Data

Panel 1 represents western blot quantitative analysis shown in Fig 7A

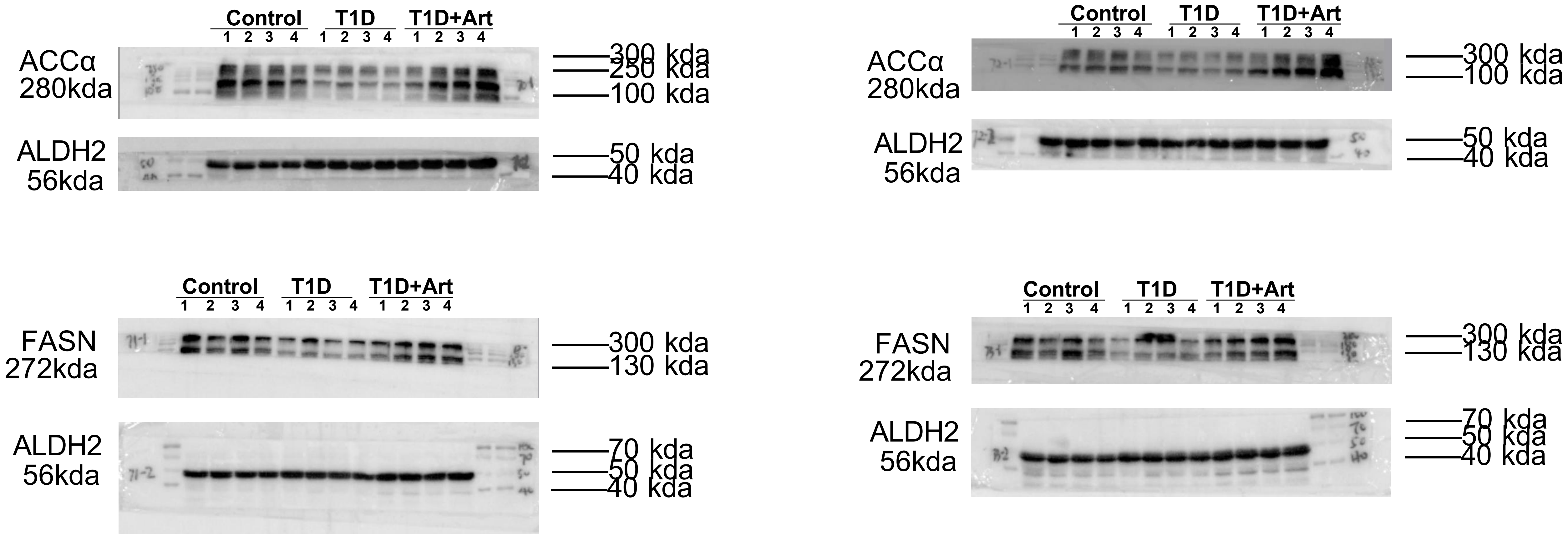

Marker: PageRulermw Prestained Protein Ladder, 40 to 300 kDa, 26625, Thermo Scientific.

Supplementary Raw Western Blot Data

Panel 1 represents western blot quantitative analysis shown in Fig 8F

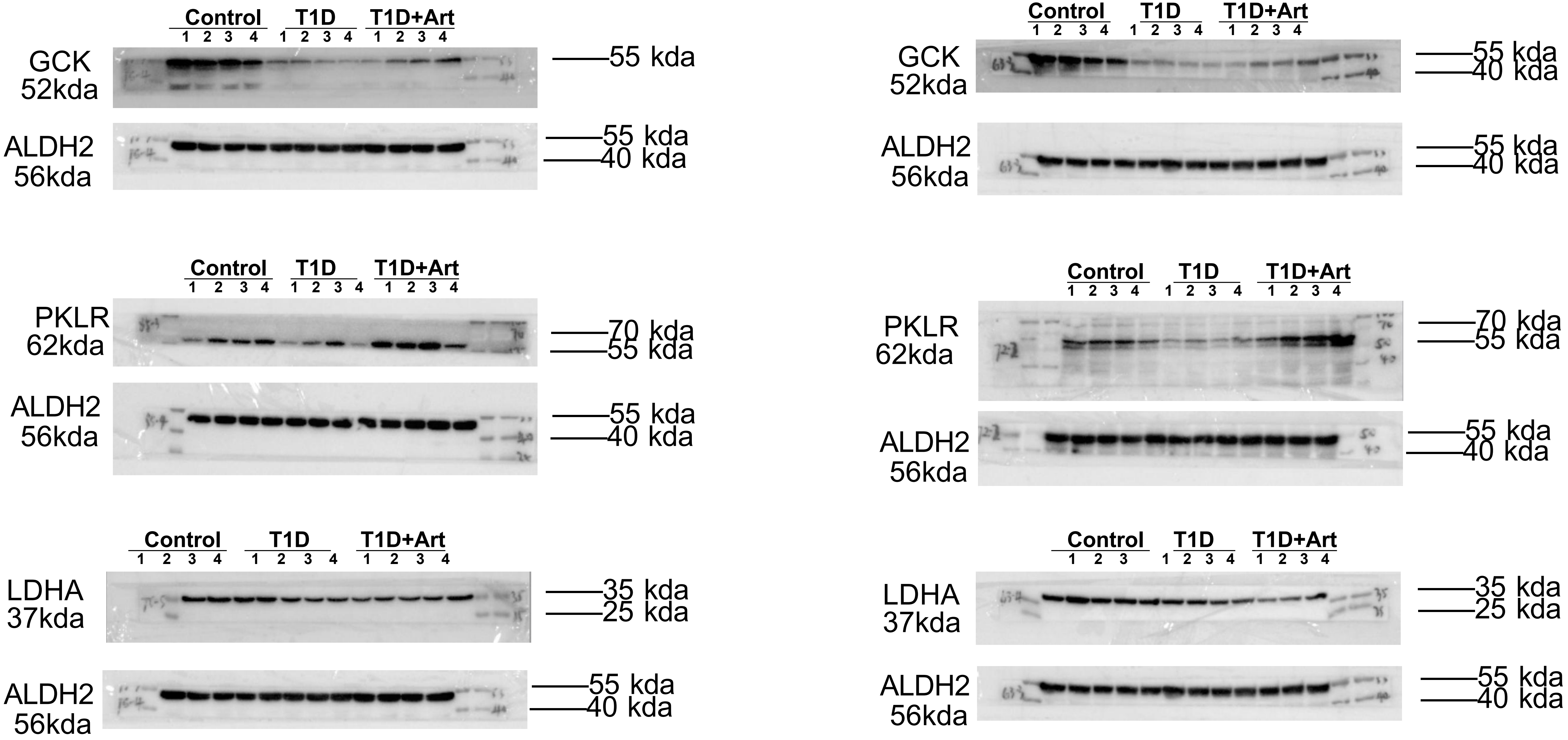

Marker: PageRuler$_{mw}$ Prestained Protein Ladder, 10 to 180 kDa, 26616, Thermo Scientific.

Supplementary Raw Western Blot Data

Panel 1 represents western blot quantitative analysis shown in Fig 8K

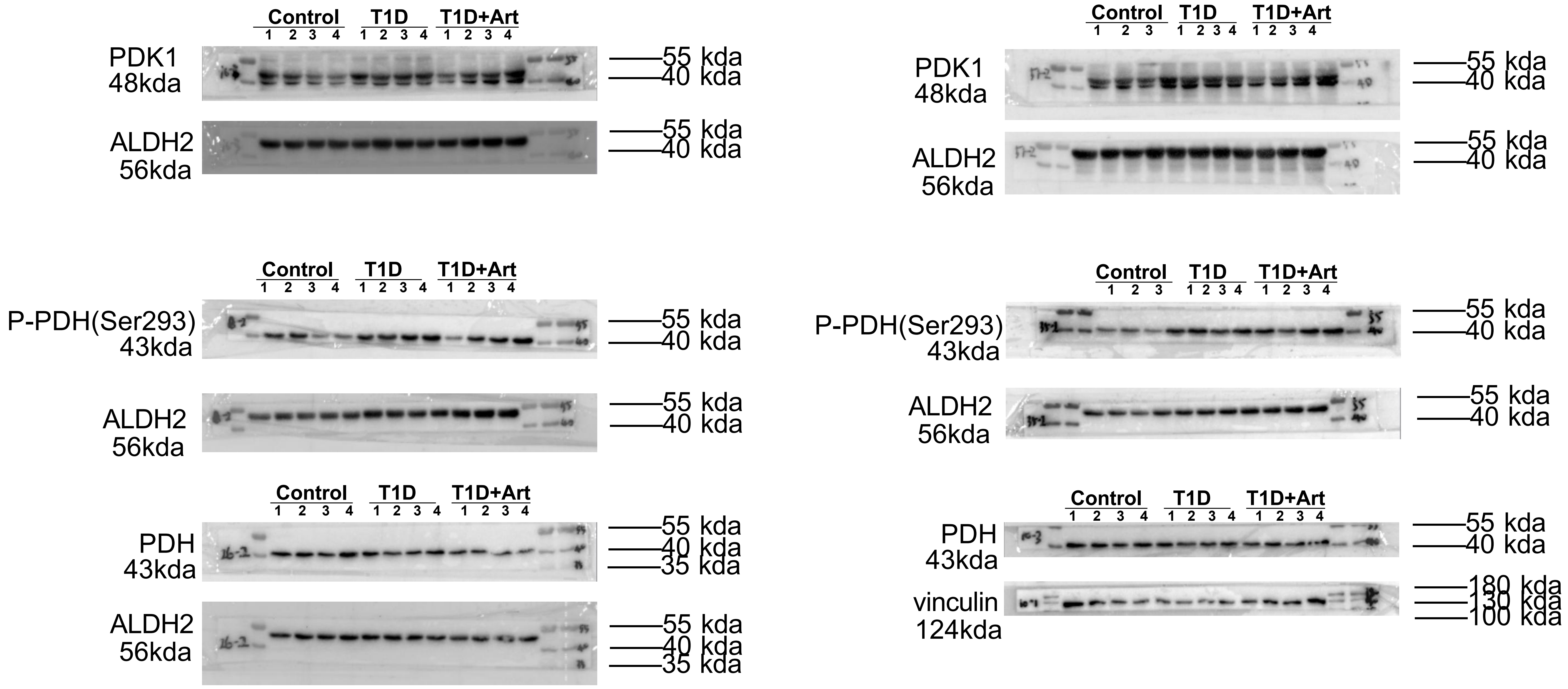

Marker: PageRuler$_{mw}$ Prestained Protein Ladder, 15 to 180 kDa, 26616, Thermo Scientific.

Supplementary Raw Western Blot Data

Panel 1 represents western blot quantitative analysis shown in Fig 9C

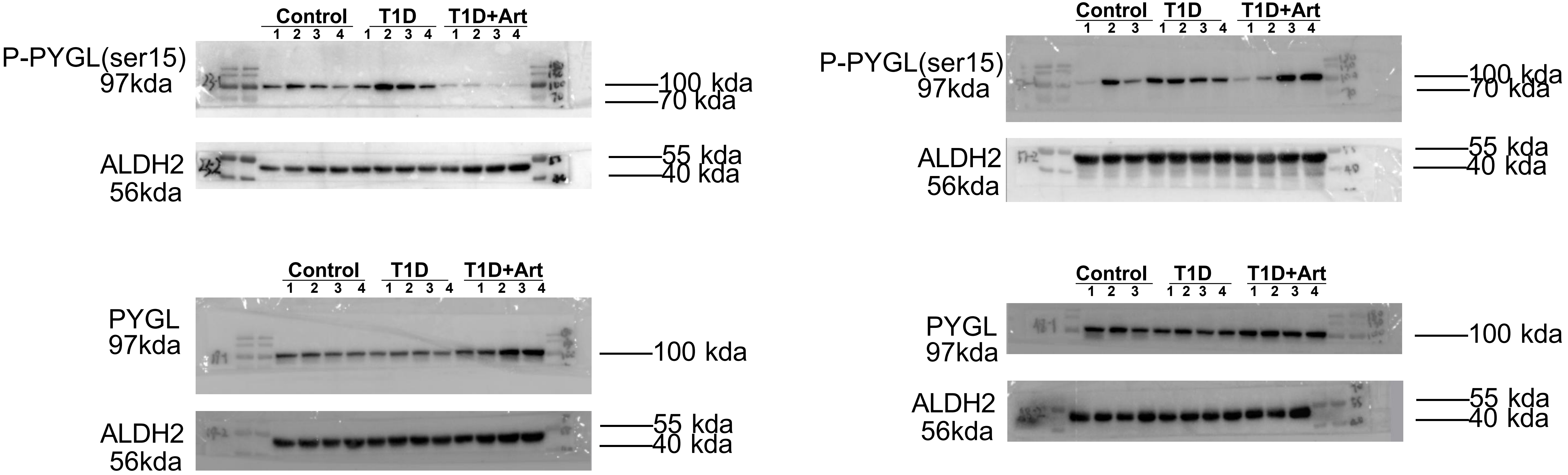

Marker: PageRuler$_{mw}$ Prestained Protein Ladder, 10 to 180 kDa, 26616, Thermo Scientific.

Supplementary Raw Western Blot Data

Panel 1 represents western blot quantitative analysis shown in Fig 9D

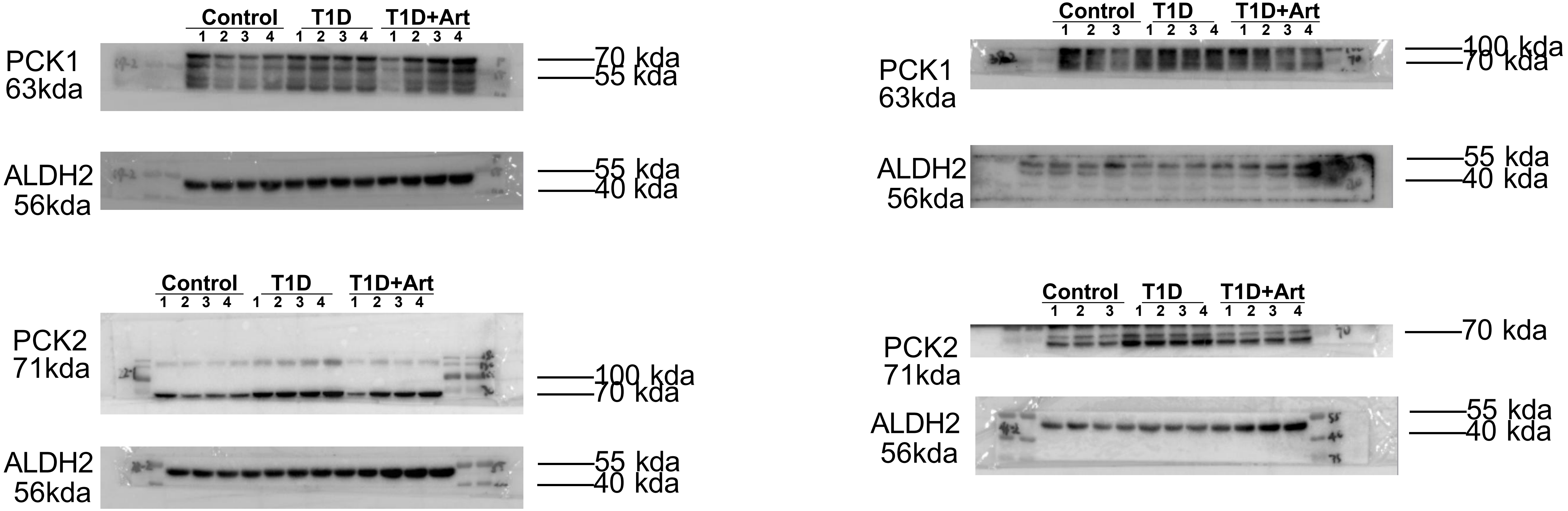

Marker: PageRuler$_{mw}$ Prestained Protein Ladder, 10 to 180 kDa, 26616, Thermo Scientific.
