# Supplementary material for: Artemether ameliorates type 1 diabetic liver injury alongside the associated defects in mitochondrial ultrastructure and central carbon metabolism
Source: PLoS One. 2026 Apr 29;21(4):e0348214. doi: 10.1371/journal.pone.0348214 (PMC13127903; doi:10.1371/journal.pone.0348214)
Supplement: S4 File — (PDF) [file pone.0348214.s004.pdf]

Fig.2C HE

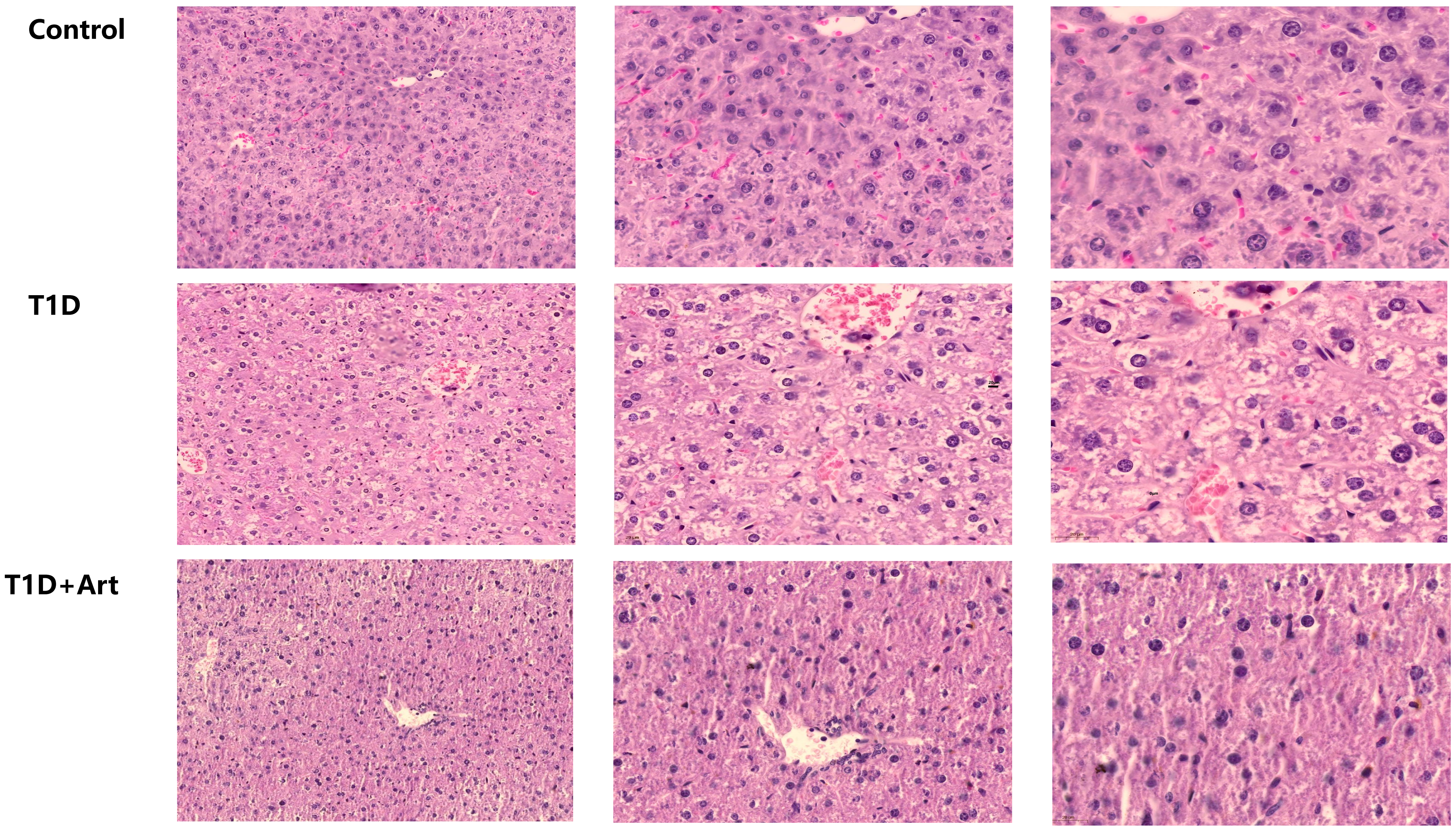

Fig.3A TEM

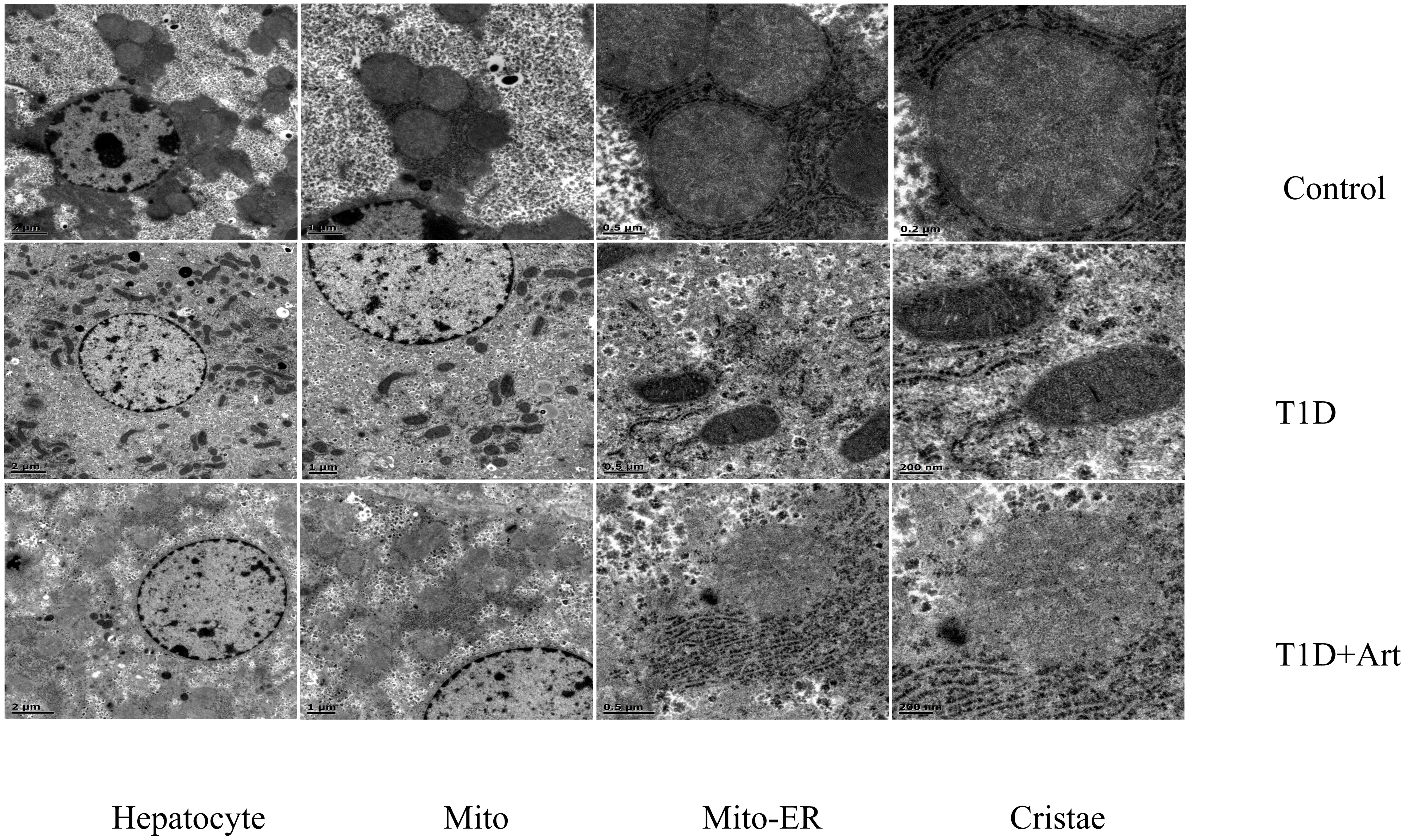
Control
T1D
T1D+Art
Hepatocyte
Mito
Mito-ER
Cristae
2 µm
1 µm
0.5 µm
0.2 µm
200 nm

Fig.4K IHC

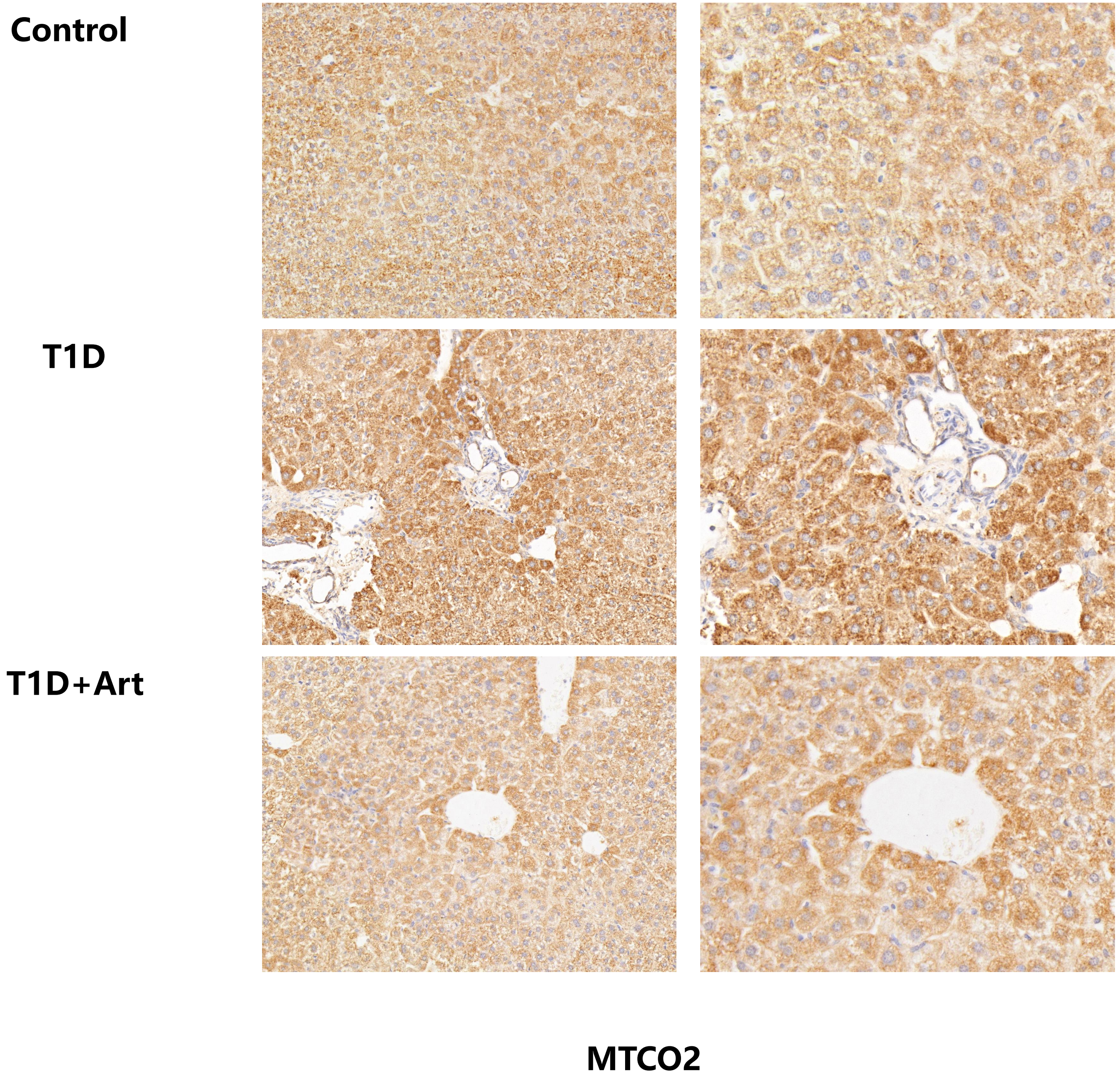

Fig.7B IHC

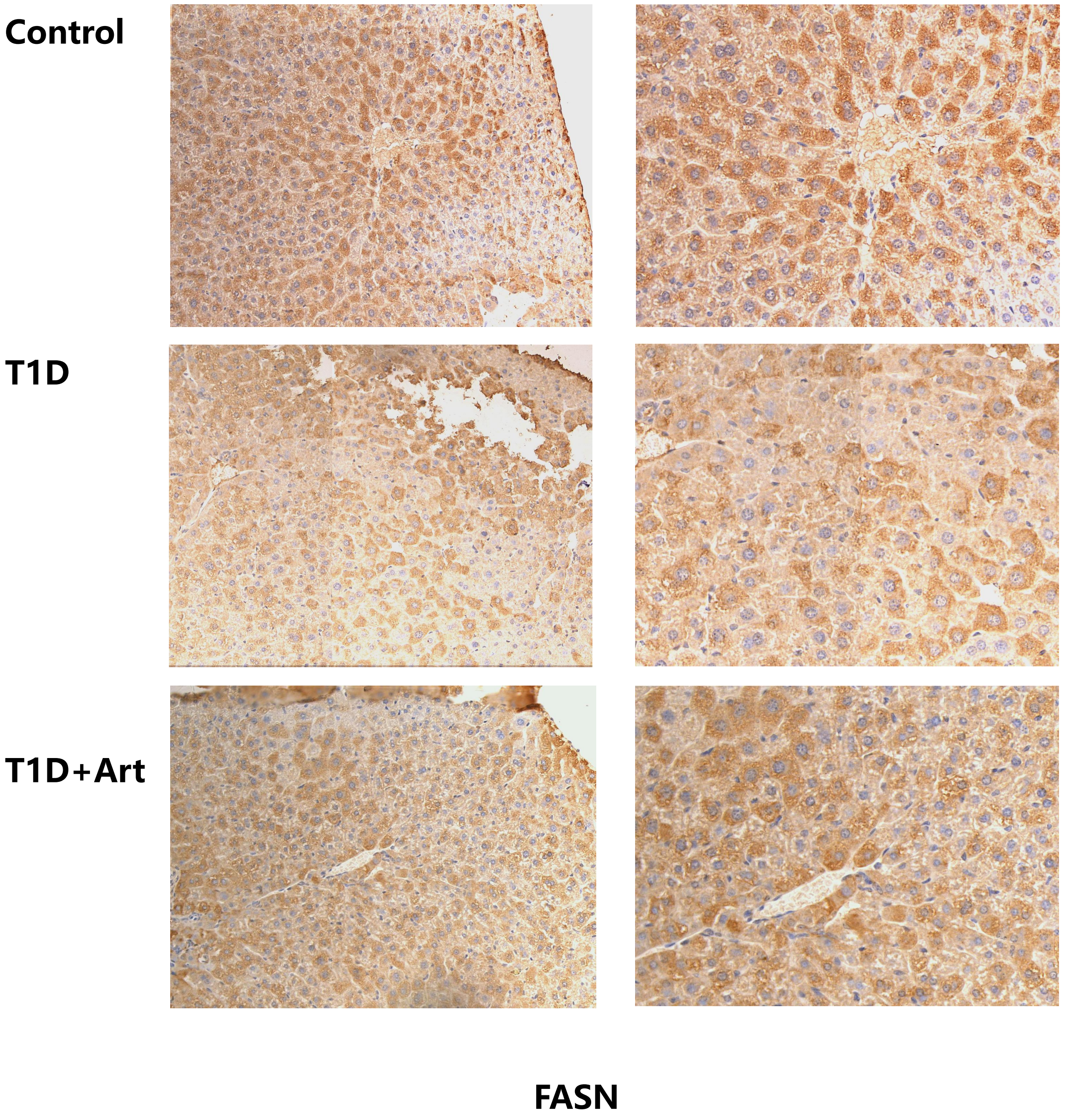

Fig.8C IHC

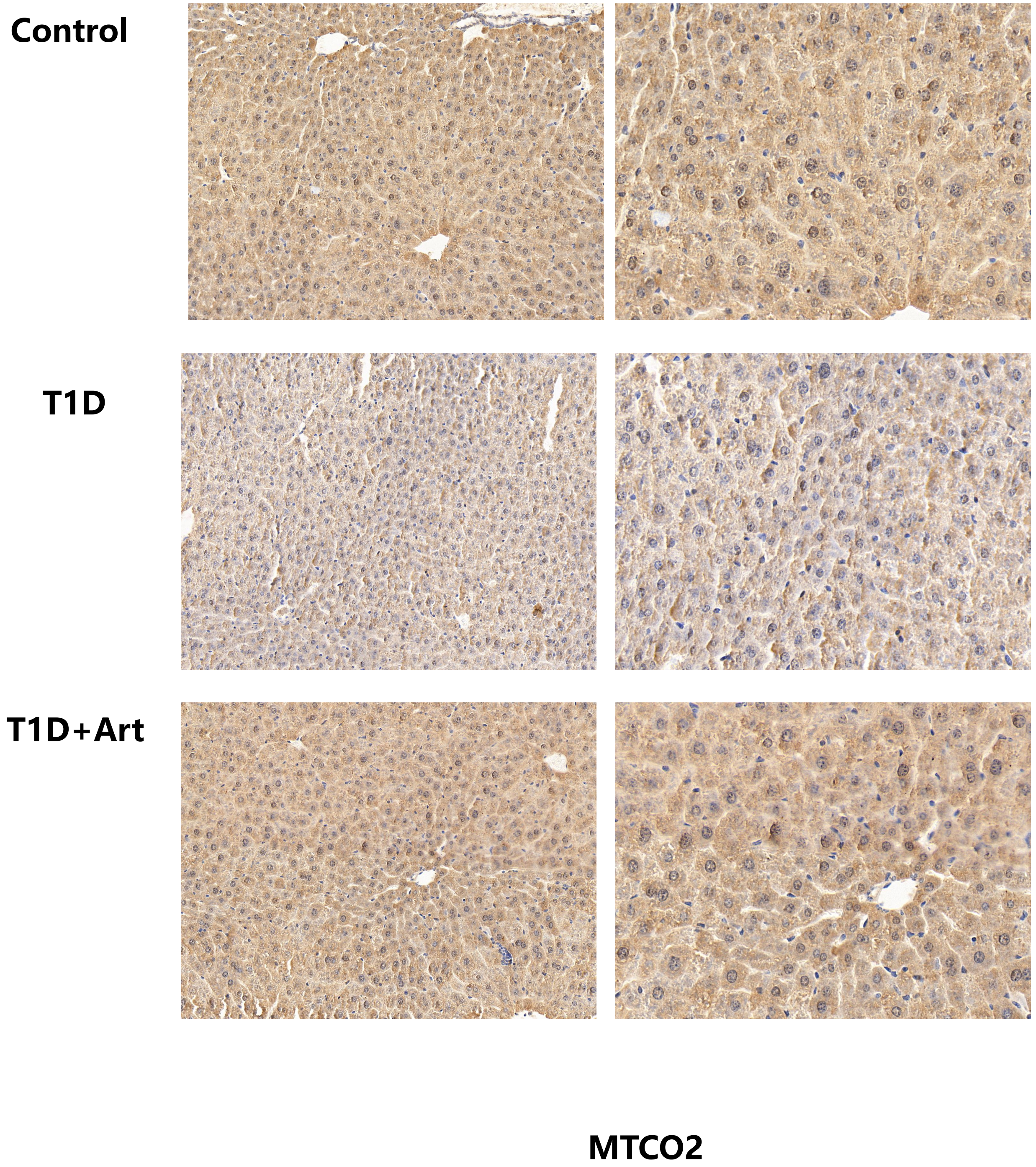

Fig.9E IHC

Control

T1D

T1D+Art

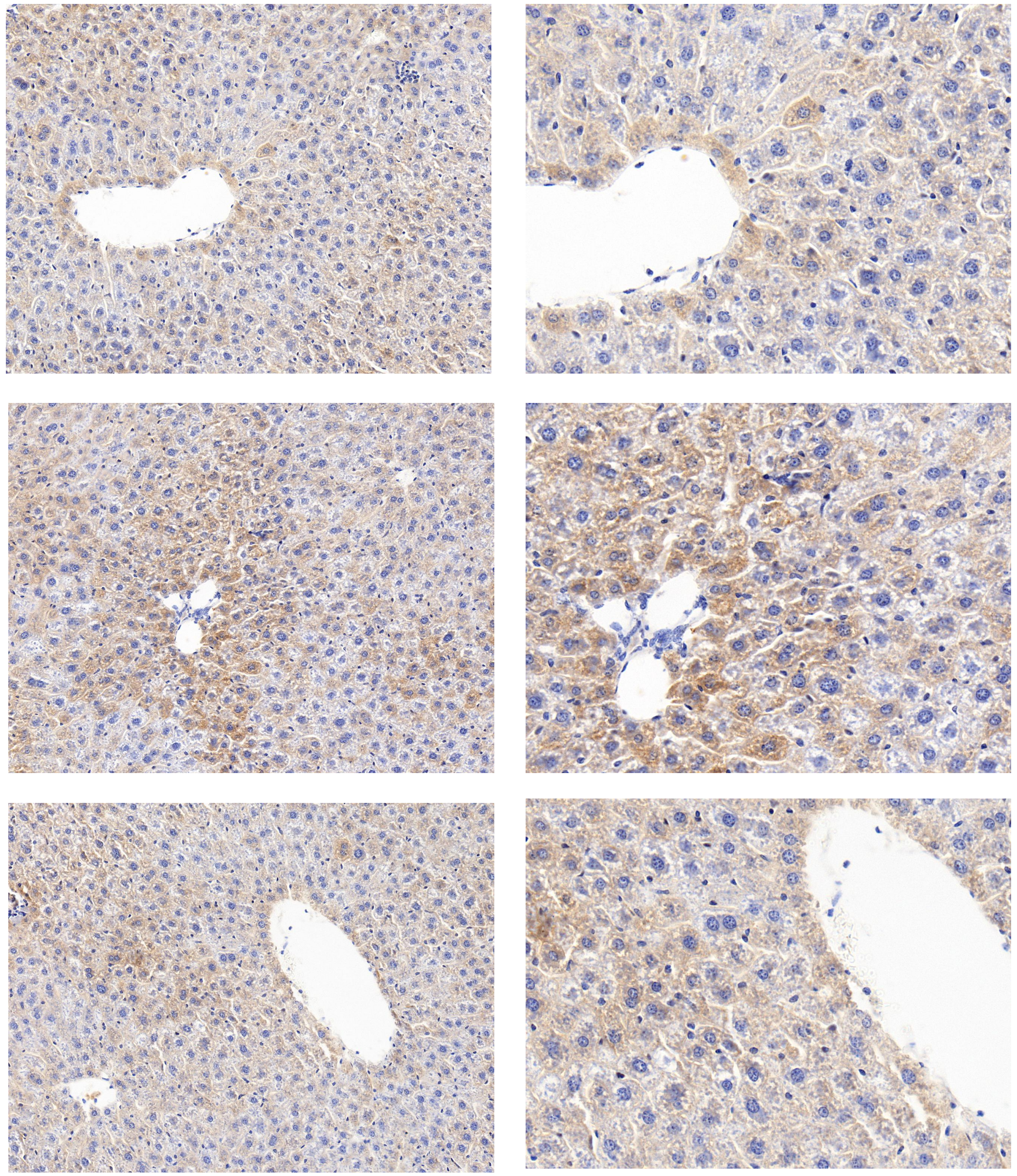

MTCO2
